# Monitoring of over-the-counter (OTC) and COVID-19 treatment drugs complement wastewater surveillance of SARS-CoV-2

**DOI:** 10.1038/s41370-023-00613-2

**Published:** 2023-12-05

**Authors:** Cheng-Shiuan Lee, Mian Wang, Deepak Nanjappa, Yi-Ta Lu, Jaymie Meliker, Sean Clouston, Christopher J. Gobler, Arjun K. Venkatesan

**Affiliations:** 1 New York State Center for Clean Water Technology, Stony Brook University, Stony Brook, NY 11794 USA; 2 Research Center for Environmental Changes, Academia Sinica, Taipei, 11529 Taiwan; 3 School of Marine and Atmospheric Sciences, Stony Brook University, Stony Brook, NY 11794 USA; 4 Department of Human Behavior, Ecology and Culture, Max Planck Institute for Evolutionary Anthropology, 04103 Leipzig, Germany; 5 Program in Public Health, Department of Family, Population & Preventive Medicine, Stony Brook University Medical Center, Stony Brook, NY 11794 USA; 6 Department of Civil and Environmental Engineering, New Jersey Institute of Technology, Newark, NJ 07102 USA

**Keywords:** COVID-19 pandemic, Wastewater-based epidemiology, Acetaminophen, Remdesivir, Bayesian models

## Abstract

**Background:**

The application of wastewater-based epidemiology to track the outbreak and prevalence of coronavirus disease (COVID-19) in communities has been tested and validated by several researchers across the globe. However, the RNA-based surveillance has its inherent limitations and uncertainties.

**Objective:**

This study aims to complement the ongoing wastewater surveillance efforts by analyzing other chemical biomarkers in wastewater to help assess community response (hospitalization and treatment) during the pandemic (2020–2021).

**Methods:**

Wastewater samples (*n* = 183) were collected from the largest wastewater treatment facility in Suffolk County, NY, USA and analyzed for COVID-19 treatment drugs (remdesivir, chloroquine, and hydroxychloroquine (HCQ)) and their human metabolites. We additionally monitored 26 pharmaceuticals including common over-the-counter (OTC) drugs. Lastly, we developed a Bayesian model that uses viral RNA, COVID-19 treatment drugs, and pharmaceuticals data to predict the confirmed COVID-19 cases within the catchment area.

**Results:**

The viral RNA levels in wastewater tracked the actual COVID-19 case numbers well as expected. COVID-19 treatment drugs were detected with varying frequency (9–100%) partly due to their instability in wastewater. We observed a significant correlation (*R* = 0.30, *p* < 0.01) between the SARS-CoV-2 genes and desethylhydroxychloroquine (DHCQ, metabolite of HCQ). Remdesivir levels peaked immediately after the Emergency Use Authorization approved by the FDA. Although, 13 out of 26 pharmaceuticals assessed were consistently detected (DF = 100%, *n* = 111), only acetaminophen was significantly correlated with viral loads, especially when the Omicron variant was dominant. The Bayesian models were capable of reproducing the temporal trend of the confirmed cases.

**Impact:**

In this study, for the first time, we measured COVID-19 treatment and pharmaceutical drugs and their metabolites in wastewater to complement ongoing COVID-19 viral RNA surveillance efforts. Our results highlighted that, although the COVID-19 treatment drugs were not very stable in wastewater, their detection matched with usage trends in the community. Acetaminophen, an OTC drug, was significantly correlated with viral loads and confirmed cases, especially when the Omicron variant was dominant. A Bayesian model was developed which could predict COVID-19 cases more accurately when incorporating other drugs data along with viral RNA levels in wastewater.

## Introduction

A new respiratory disease, COVID-19 (coronavirus disease 2019), caused by severe acute respiratory syndrome coronavirus 2 (SARS-CoV-2) was first identified in Wuhan, China, in December 2019. COVID-19 then rapidly spread to the rest of the world and its outbreak was declared a pandemic by the World Health Organization (WHO) on March 11, 2020. The disease spreads primarily via air transmission, such as droplets and aerosols containing the virus from infected people when they cough, sneeze, speak, and breathe [1, 2]. People infected with COVID-19 experience variable symptoms from mild to severe illness. Since gastrointestinal symptoms are also reported, studies have revealed that the virus can be found in the feces of COVID-19 patients, and the shedding of the virus in feces lasts longer than those from the respiratory tract, regardless of whether it is infectious or not [3–5]. Therefore, the signal of the virus in sewage can be an informative tool to monitor the prevalence of COVID-19 in communities, providing an early indication for potential outbreaks.

Wastewater-based epidemiology (WBE) is a relatively new field that began to develop in the early 2000s and has achieved huge developments in recent years, using wastewater monitoring of chemicals (e.g., illicit drug use), biomarkers, and pathogens to acquire qualitative and/or quantitative data on the lifestyle and health of people living within a catchment area [6–13]. It can provide near-real-time monitoring, covering both spatial and temporal trends while respecting the privacy of individuals and yielding information applicable to entire populations. It can have some disadvantages, for example, lack of representative selection and stability of some biochemical indicators in sewage, and uncertainties with regard to wastewater flows and the daily variation of the overall population contributing to wastewater in the catchment [14, 15]. Nevertheless, it can still serve as a complementary approach to current infectious disease surveillance techniques [12, 16], and the U.S. Centers for Disease Control and Prevention (CDC) launched a nationwide wastewater surveillance system in September 2020 to monitor SARS-CoV-2 in sewage, obtaining community-wide information with respect to the prevalence and temporal trends of COVID-19 [17]. Such massive cumulative data is valuable for implementing WBE against COVID-19 or other possible infectious diseases in the future.

Since the global outbreak of COVID-19, many studies have used WBE for COVID-19 surveillance, correlating the virus detected in sewage with reported cases, such as the U.S., Canada, the Netherlands, India, and France [18–25]. These studies further calculate the lead time of SARS-CoV-2 in wastewater prior to COVID-19 prevalence in the communities, which ranges from 0 to 14 days, suggesting the potential effectiveness of using WBE as a qualitative early warning system for the COVID-19 outbreak. Furthermore, attempts to forecast community-level COVID-19 prevalence (e.g., case count prediction) using WBE data with modeling or machine learning techniques were made and a few results claim that the prediction could be made up to 3 weeks in advance [26–29]. However, most of the wastewater surveillance works rely on correlation analysis and show overlapped trends of COVID-19 cases and virus/drug concentrations, with or without time lags [18, 19, 22, 30]. Olesen et al. [31] provide a comprehensive summary of these existing studies, their applications, and limitations. In contrast to what has been pointed out by Olesen et al. [31], however, those studies are qualitative in the sense that they provide correlations or lead times of surge between virus-drug concentrations and COVID-19 cases without a formal transformation between the two. They are useful for the early warning of epidemic outbreaks, but one cannot derive a reasonable range of confirmed cases from the detected concentrations of the substances. Of the models capable of predicting confirmed cases quantitatively, either the forecasting periods are short [26], or the prediction errors are large [32]. For example, McMahan et al. [32] develop a Susceptible-Exposed-Infectious-Recovered (SEIR) model to capture the number of infections. They found that the ratio of actual infections to confirmed cases aligns well with previously reported data. However, the predicted cases of COVID-19 infections by virus RNA (ribonucleic acid) copies have large confidence intervals, ranging from 500 to 1500 individuals. In that case, the performance and accuracy of modeling need to improve further with long-term and high-throughput surveillance data, and could be assisted with other pharmaceuticals and biomarkers such as over-the-counter (OTC) and COVID-19 treatment drugs in sewage [30, 33].

People infected by COVID-19 would often experience common cold-like symptoms including cough, headache, and fever, when the viruses begin to influence their bodies. In addition to seeking PCR testing or performing rapid antigen testing, self-medication with OTC drugs is likely to be people’s first action to mitigate their illness. A patient with severe symptoms would be treated with COVID-19 treatment drugs in the hospital. These pharmaceuticals would be excreted in patients’ urine and then into sewage. Therefore, the detection of these pharmaceuticals in wastewater influent may provide an early signal for the COVID-19 outbreak in a community and complement ongoing SARS-CoV-2 RNA-based measurements. For example, antiviral drugs such as chloroquine, hydroxychloroquine, and remdesivir were authorized for emergency use by the FDA on March 28, 2020, and May 1, 2020, respectively [34, 35]. Although the FDA revoked emergency use authorization (EUA) for chloroquine and hydroxychloroquine later in June 2020 [36], these two drugs were still used in hospitals, treating prophylaxis of malaria and autoimmune diseases such as rheumatoid arthritis and systemic lupus erythematosus [37].

The goal of this paper was to identify complementary chemical biomarkers that correlate with SARS-CoV-2 RNA levels in raw sewage. Using solid-phase extraction (SPE) followed by mass spectrometry for the analysis of chemical biomarkers can be faster, more precise, and more sensitive than RNA analysis, primarily due to the stability concerns of RNA viruses in the sewage matrix. Hence, the study results may offer insights into identifying abundant chemical indicators to complement the ongoing wastewater surveillance efforts across the globe. In this study, we measured SARS-CoV-2 RNA in raw sewage from a municipal wastewater treatment plant (WWTP) on Long Island, New York, from June 2020 to January 2022 (~20 months), crossing different periods of various dominant viral lineages. In addition to the viral data, we also quantified concentrations of COVID-19 treatment drugs and other OTC drugs simultaneously.

## Materials and methods

### Wastewater sampling

Wastewater samples were collected from a WWTP located in Suffolk County, NY. The WWTP serves ~330,000 people in its sewer catchment and treats ~30.5 million gallons of wastewater daily. Untreated raw sewage influent was collected via autosampler every 15 min to make up a 24-h composite sample, refrigerated upon collection to <6 °C. The 24-h composite sample was subsampled into a 500-ml polypropylene bottle, stored in a cooler with ice packs, and then transferred to our lab (~1 h drive) for subsequent analyses. Viral analysis was performed immediately upon sample receipt and the entire procedure was completed within a day. The remaining sample was then split into two aliquots and stored at −80 °C without adding any preservatives, one for drug analysis and the other as an archived sample. Suspended particles in the samples for drug analysis were removed through vacuum filtration (1 µm glass fiber) prior to freezing. The subsequent drug analysis was performed within 3 weeks. Sampling was initiated on June 2020, with daily sampling from June 3 to June 9, weekly sampling from June 9 to July 7, and biweekly sampling from July 7 to December 22, 2020, and twice weekly sampling from January 2021 through January 6, 2022.

### Detection and quantification of SARS-CoV-2 RNA

Twenty-four-hour composite samples of raw sewage were centrifuged at 4200 rpm for 30 min at 4 °C in order to remove large particles and debris before polyethylene glycol (PEG) precipitation. To evaluate the viral recovery rates from wastewater, bovine coronavirus (BCoV), which belongs to the same genus as SARS-CoV-2, was spiked into the supernatant. The viral particles in 40 ml of samples were precipitated with PEG 8000 (Millipore Sigma, Burlington, MA) and NaCl (5 M, Millipore Sigma, Burlington, MA) and then incubated overnight at 4 °C. RNA from the PEG-precipitated wastewater was extracted by Qiagen QIAamp DSP viral RNA mini kit (Qiagen, Hilden, Germany) according to manufacturer’s instructions and eluted in 100 µl by nuclease-free water. The concentrations of RNA were measured by NanoDrop One Spectrophotometer (Thermo Fisher Scientific, Waltham, MA). All RNA samples were stored at −80 °C and subjected to cDNA synthesis within the same day of RNA extraction to avoid losses associated with storing and freezing and thawing RNA extracts.

Reverse transcription was performed by High Capacity RNA-to-cDNA Kit (Applied Biosystems, Waltham, MA) at 37 °C for 60 min, and stored at −20 °C until further analysis. The cycling condition was 95 °C for 10 min, followed by 40 cycles of 95 °C for 5 s and 55 °C for 40 s, and 98 °C for 10 min. The total volume of each reaction was 14.5 µl containing 7.25 µl of QuantStudio 3D Digital PCR Master mix v2 (Applied Biosystems, Massachusetts, USA), 0.725 µl of primer and probe (N1/ BCoV), 0.725 µl of TaqMan® Copy Number Reference Assay RNase P (as an internal control, Applied Biosystems, Waltham, MA), 4.8 µl of nuclease-free water, and 1 µl of cDNA template. Digital PCR was performed using N1 primers and probe set from 2019-nCoV RUO Kit (IDT # 10006713) with the CDC-recommended sequence and BCoV set against the BCoV gene as an external reference on a QuantStudio 3D Digital PCR (Applied Biosystems, Massachusetts, USA). Nuclease-free water was used as non-template control (NTC) and plasmids containing the complete nucleocapsid gene from 2019-nCoV (IDT # 10006625) were used as a positive control. Data analysis was performed with the online version of the QuantStudio 3D AnalysisSuite Cloud Software. The limit of detection for the N1 gene was 1.4 copies/reaction.

### Detection of COVID-19 treatment drugs and other pharmaceuticals

Standards of COVID-19 treatment drugs (i.e., remdesivir, chloroquine, and hydroxychloroquine), other pharmaceuticals (OTC drugs), and their corresponding isotopically labeled compounds were purchased from Toronto Research Chemicals Inc (Ontario, Canada), Sigma-Aldrich (MO, USA), Fisher Scientific (MA, USA), Cerilliant (TX, USA), CDN Isotopes (Quebec, Canada), and Cambridge Isotope Laboratories (MA, USA). A list of all compounds used in this study is presented in Table S1. The 26 pharmaceuticals, including OTC drugs and the metabolites, were chosen because of their relatively high prescriptions per population in the U.S. and high environmental detection frequencies in previous studies [38, 39].

Due to the low concentration of COVID-19 treatment drugs, SPE was required to concentrate the sample for detection. In contrast, other pharmaceuticals were measured by direct injection after dilution. In brief, a 100-ml sample was transferred out and spiked with a surrogate standard (hydroxychloroquine-D4) to trace the extraction yield prior to SPE. After conditioning the SPE cartridge (Waters Oasis HLB, 200 mg, 6 cc) with methanol and deionized water, the whole sample was loaded onto the cartridge, and after which the cartridge was eluted sequentially with 4 ml methanol, resulting in ~25-fold preconcentration. Extracts were stored at −20 °C until analysis. Prior to analysis, the extract was diluted with deionized water (50:50 MeOH: H_2_O) and spiked with the internal standard. For other pharmaceuticals, a 100-µl sample was taken out and diluted 10-times with deionized water and methanol to constitute a final concentration of 10% methanol. The isotopically labeled internal standards were then added before analysis. The detailed information for the surrogates and internal standards is shown in Table S1.

Detection and quantification of the target compounds in extracts were carried out using an Agilent 6495B triple-quadrupole mass spectrometer (LC-MS/MS) with an electrospray ionization source in positive ion mode (ESI+), using Multiple Reaction Monitoring (MRM) to monitor the precursor ions and product ions (Table S1). The detailed instrumental conditions are shown in Table S2.

### Stability of COVID-19 treatment and OTC drugs in wastewater

The wastewater temperature in underground sewer pipes in the study area was measured to vary between 10–12 °C in winter and 18–20 °C in summer, as provided by wastewater operators. The travel time of sewage from houses to the WWTP in the study area ranged from 40 min to 8 h, depending on the distance. Additionally, the collected wastewater sample could reside in the composite sampler for up to 24 h at 6 °C. Thus, we performed a controlled experiment to assess the stability of the analytes of interest at different temperatures within 24 h. In brief, a suite of COVID-19 treatment and OTC drugs was spiked (50 ng each) into 50 ml of unfiltered raw wastewater. The spiked wastewater samples were stored at 4, 12, and 20 °C. Each temperature had triplicate samples. At *t* = 0 h and *t* = 24 h, 5 ml aliquot was taken out for analysis and followed the sample preparation procedure described above for LC-MS/MS analysis.

### COVID-19 cases

New reported confirmed cases of COVID-19 were recorded by the Suffolk County Department of Health, NY. Data at the zip code level were shared with our research team to support COVID-19 research in the region. We identified the 13 zip codes in the catchment area of the WWTP and summed the number of cases daily in the catchment area to create a 7-day rolling average number of cases.

We also received reports from Stony Brook University Hospital of daily hospitalized cases, and milligram of COVID-19 treatment drugs, hydroxychloroquine and remdesivir prescribed daily beginning Oct 3, 2020 to the present. This hospital is not physically located in the catchment area but is the closest level 1 Trauma center to the catchment area, and receives patients from the catchment area. These data, therefore, are not used as proxies for the amount of remdesivir or hydroxychloroquine in the catchment area, but rather are useful for understanding temporal trends in prescriptions of these treatment drugs in the region. As shown in Fig. S1, remdesivir usage can reflect the case trend in the hospital, whereas hydroxychloroquine usage remains relatively stable over time.

### Population correction

Estimating the actual population contributing to sewage flow during the sampling period is challenging but is essential as it directly influences the concentration of biomarkers in wastewater. Several endogenous and exogenous human biomarkers have been proposed to serve as a tool for population normalization [16, 40]. In this study, we selected caffeine, a stimulant excreted in human urine, to estimate the serviced population in the WWTP sewershed because its level in wastewater is known to be stable and features <10% degradation within 24 h as shown in our preliminary experiment (Fig. S2). Over the ~20-month period, the caffeine concentration in the samples showed little variation over time with a mean concentration of 88.2 ± 20 µg/l (range: 48.2–148 µg/l). Time series data of SARS-CoV-2 RNA, COVID-19 treatment drugs, and other pharmaceuticals were normalized by caffeine using the equation below:

1 $$\frac{{[{{{{{{\rm{Virus}}}}}}}\,{{{{{{\rm{or}}}}}}}\,{{{{{{\rm{drug}}}}}}}]}_{t}}{\frac{{[{{{{{{\rm{Caffeine}}}}}}}]}_{t}}{{[{{{{{{\rm{Caffeine}}}}}}}]}_{{{{{{{\rm{avg}}}}}}}}}}$$

where [Virus or drug]_*t*_ is the virus or drug concentration at time = *t*, [Caffeine]_*t*_ is the caffeine concentration at time = *t*, and [Caffeine]_avg_ is the average caffeine concentration.

### Model development

We developed several Bayesian models to predict confirmed cases with concentrations of virus gene copies and other biomarkers in wastewater samples. The Bayesian framework, as compared to classical statistics, allows us to update our current models with future data collection. Except for the confirmed cases, all other variables were adjusted in the following ways for ease of modeling or interpretation. First, virus concentration was log-transformed with base 10. All other measured chemicals were divided by their maximum value in the sample and rescaled into values between 0 and 1. This way, we retained zero as a reference point while being able to make sense of the priors across variables in our modeling. In order to select our variables of interest to fit the model, data exploration was performed and described in the Supplementary Information.

We regressed our dependent variable, the confirmed cases at time *t* (*C*_*t*_), on lags of the predictor variable(s) (*X*_1*, t*−1_, *X*_1, *t−2*_, *X*_1, *t*−3_*,*…; *X*_2, *t*−1_, *X*_2, *t*−2_, *X*_2, *t*−3_,…; *X*_3, *t*−1_, *X*_3, *t*–2_, *X*_3, *t*−3_,…, etc.,), via a generalized linear model (GLM). The number of confirmed cases was modeled as a binomial distribution with the trial number equal to the population in the sewershed area (*N* = 3.3 × 10^5^). The probability of individual infection is a logistic function of linear combinations of our predictor variables. The models have a general form:

2 $${C}_{t} \sim {{{{{{\rm{Binominal}}}}}}}\left(N,p\right)$$

3 $${{{{{{\rm{logit}}}}}}}\left({p}\right)=\alpha +\mathop{\sum }\limits_{{i}=1}^{m}\left(\mathop{\sum }\limits_{{j}=1}^{n}{\beta }_{{ij}}{X}_{i,t-j}\right)$$

4 $$\alpha \sim {{{{{{\rm{Normal}}}}}}}\left(\bar{\alpha },{\sigma }_{\alpha }\right)$$

5 $${\beta }_{{ij}} \sim {{{{{{\rm{Normal}}}}}}}\left({\bar{\beta }}_{{ij}},{\sigma }_{{\beta }_{{ij}}}\right)$$

where *m* = number of substances (e.g., viral or/and chemical concentrations) and *n* = number of lags (observations before the focal day) used for prediction. We considered three specific sets of models. For each set of models, we tested possible combinations of variables according to Data Exploration and Cross Correlation (see SI). The choice of priors was examined by predictive simulations in the SI, and the posterior distributions of parameters were estimated using Markov Chain Monte Carlo (MCMC). We retained the model with the best predictive performance based on the Watanabe–Akaike Information Criterion (WAIC). Two general rules were also applied in the modeling. First, we used consecutive lags because the change of predictor variables was more likely to have gradual effects on our outcome variable. Second, to avoid overfitting, we maintained at least ten observations for each predictor included in the models, as 111 observations were present in our sample.

## Results and discussion

### Detection of SARS-CoV-2 RNA, COVID-19 treatment drugs, and other pharmaceuticals

From June 2020 to May 2022, over ~24 months of sampling and monitoring (*n* = 183), over 99% of the samples contained detectable SARS-CoV-2 RNA, ranging from 10^2^ to 10^6^ gene copies/l. The apparent temporal trend of virus concentrations in wastewater was very similar to the confirmed cases in the catchment area (Fig. S3). The detected viral data was able to capture the winter spike in 2020 of Alpha-Epsilon variants, Delta-variant wave, and Omicron-variant surge, confirming the feasibility of analyzing viruses in wastewater to track their spread. More detailed analyses are presented in the following sections.

For COVID-19 treatment drugs (data spanned from June 2020 to Jan 2022), hydroxychloroquine was detected in 100% of the samples (*n* = 111). High detection frequencies were also found for its metabolites, desethylchloroquine (99%) and desethylhydroxychloroquine (93%). In contrast, chloroquine was barely detected (9%), and remdesivir was only detected in approximately one quarter of the samples (23%). We suspected that the low detection frequency of chloroquine and remdesivir was due to their instability in wastewater, as can be the case for many compounds [41, 42]. Our stability experiments showed that ~40% of chloroquine and remdesivir was lost from wastewater at 4 °C after 24 h (Fig. S2). At 20 °C, a significant loss was observed for remdesivir (~87%) in a day, followed by chloroquine (~58%) and desethylchloroquine (~58%). These results partially explain why remdesivir was not frequently detected in the wastewater samples. The temperature also had a significant effect on the detection of SARS-CoV-2 RNA in wastewater. In winter, the viral titer was able to be detected up to 100 h after shedding in wastewater but it was reduced to 20 h in summer [43]. The variation of temperature affects the degradation rates of RNA, thus reducing the detectability of SARS-CoV-2 RNA despite equal initial loading.

Beyond antiviral drugs, 13 out of 26 pharmaceuticals assessed were detected (DF = 100%, *n* = 111) in all wastewater samples during this period. Acetaminophen (mean = 83.6 µg/l, range: 19.5–237 µg/l) and caffeine (mean = 88.2 µg/l, range: 48.2–148 µg/l) and its metabolite paraxanthine (mean = 23.1 µg/l, range: 12.6–37.8 µg/l) were the most abundant chemicals in wastewater samples. A summary of statistics of the viral RNA and all the drugs measured in this study is listed in Table S3.

### Correlations and temporal trend of viral genes and biomarkers in sewage samples

**Fig. 1:**
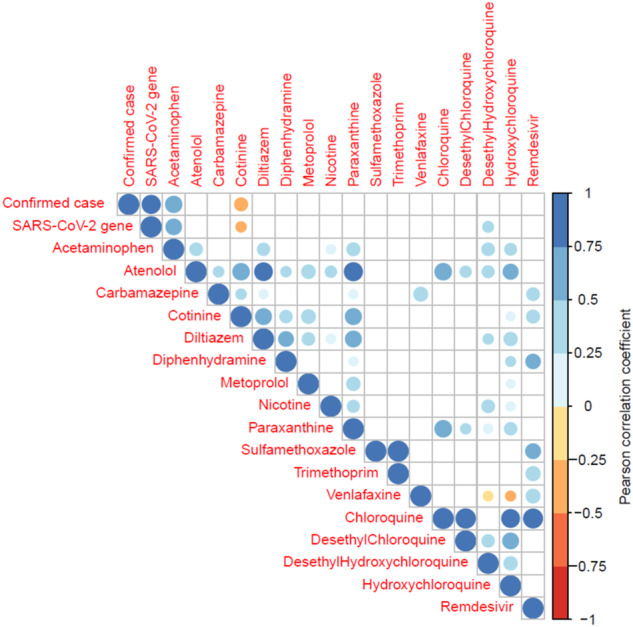
Summary of the Pearson correlation coefficient (*R*) between variables (*n* = 111). Only significant correlations (*p* < 0.05) were shown. All the variables were normalized by caffeine, except for reported confirmed cases.

In this section, raw data of viral and chemical concentrations were normalized by caffeine (Eq. (1)) to compare with the confirmed cases for correlations and/or temporal trend similarities. Figure 1 shows a summary of the Pearson correlation between all the reported and measured variables in this study (colored circles shown if *p* < 0.05). As shown in the figure, our SARS-CoV-2 data showed a very strong correlation (*R* = 0.81, *p* < 0.01) with the confirmed cases. The confirmed cases also positively correlated with acetaminophen (*R* = 0.66, *p* < 0.01), presumably due to its use for relieving symptoms induced by COVID-19. Interestingly, cotinine, a primary metabolite of nicotine, was negatively correlated with the confirmed cases (*R* = −0.42, *p* < 0.01). As a few studies claimed that smoking can depress pulmonary immune function and therefore favored progression of COVID-19 [44–46], a decline of cotinine in wastewater may reflect a decrease in smoking due to the higher possibilities of infection and hospitalization of smokers in the sewer catchment area. Nevertheless, further investigation with more evidence is needed to elucidate the reasons for this observed trend. Among COVID-treatment drugs, hydroxychloroquine was positively correlated with its known metabolites desethylchloroquine and desethylhydroxychloroquine. It should be noted that the detection frequency of chloroquine was low, and therefore its calculated correlation with other variables relied on few data points and was not representative.

**Fig. 2:**
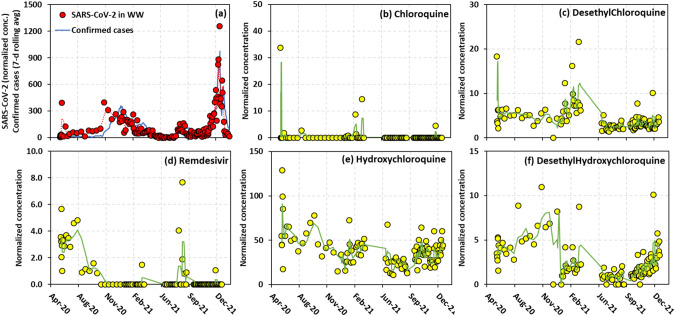
Temporal trend of SARS-CoV-2 virus and COVID-treatment drugs in the sewer catchment area from June 2020 to January 2022. **a** shows the daily reported confirmed cases and the normalized virus concentration in WW over time. **b**–**f** are the normalized concentrations of COVID-treatment drugs and their metabolites detected in WW over time. The green line in (**b**–**f**) represents a moving average trendline.

Figure 2 shows the time series of confirmed cases in the sewershed and caffeine-normalized SARS-CoV-2 and COVID-treatment drugs. RNA analysis tracked the confirmed cases in the sewershed very well throughout the entire 20-month period, successfully capturing the second wave of COVID-19 in New York from late October 2020 to early May 2021, a minor third wave from mid-July 2021 to October 2021, and finally the surge of the Omicron variant of COVID-19 starting from November 2021 (Fig. 2a). Consistent with the COVID-19 trends, the SARS-CoV-2 concentrations varied, increasing from mid-October 2020 and reaching a peak in late December 2020. The concentrations remained comparatively steady between June and mid-July 2020, following a rapid increase in early August 2020 and reaching a peak by the end of the month. This brought the third wave of COVID-19. Subsequently, the SARS-CoV-2 RNA kept increasing and climbed to a maximum concentration during the 20-month study in early January 2022, which preceded the Omicron surge. The lead time of the virus data over the confirmed cases was about 3–4 days, estimated by the maximum Pearson correlation. The lead time was comparable to the lead time of 0–6 days reported in several previous WBE studies [5, 18–21, 25]. A few studies reported a longer lead time, such as 14 days which could provide sufficient time to inform public health actions from a study in Gujarat, India [47], 5–8 days from studies in Paris, France [24], Bozeman, MT, USA [21], and New Haven, CT, USA [22]. It is without a doubt that WBE can provide early-warning capability, but the calculated lead time can vary considerably among different study sites. It can be attributed to sampling methods, sewer system configurations, sanitation facilities, and climate and weather conditions that could influence the analysis of viral copies of SARS-CoV-2 RNA in samples. Thus, Kumar et al. [43] concluded that WBE can be applied better in countries having proper water, sanitation, and health conditions, as well as well-connected sewer systems. Lastly, the lead time is relative to confirmed cases that can be reported in a timely manner, and the accurate confirmed cases rely on the efficiency and capacity of COVID-19 testing, which could vary between communities.

The temporal trend of hydroxychloroquine was similar to desethylchloroquine and desethylhydroxychloroquine (Fig. 2), suggesting that the latter two compounds were derived from hydroxychloroquine [48]. As mentioned above, hydroxychloroquine has still been used to treat COVID-19, although its EUA was revoked in June 2020. While there were not any significant correlations between hydroxychloroquine and the viral loads in wastewater or confirmed cases over time (Fig. 1), there was a statistically significant correlation (*R* = 0.30, *p* < 0.01) found between the SARS-CoV-2 genes and desethylhydroxychloroquine. The peaks of desethylhydroxychloroquine in November 2020 and December 2021 match the increase in the SARS-CoV-2 genes in the same periods (Fig. 2f). Remdesivir is a drug that was specifically prescribed for COVID-19 treatment, and we found two peaks during the monitoring period. The first peak appeared right after the EUA of remdesivir, and the latter corresponded to the rise of confirmed cases in September 2021. The overall trend of remdesivir concentrations, however, was not similar to either the viral data or the confirmed cases perhaps due to its instability in wastewater.

**Fig. 3:**
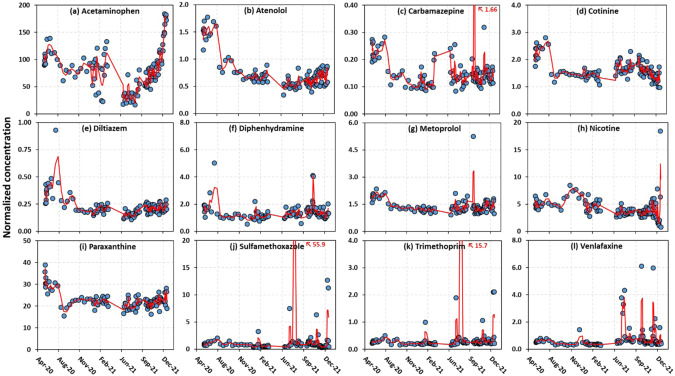
Temporal trend of pharmaceuticals in the sewer catchment area from June 2020 to January 2022. The normalized concentrations of twelve pharmaceuticals with 100% detection frequency versus time are shown in (**a**–**l**). All the concentrations are normalized to caffeine. The red line in (**a**–**l**) represents a moving average trendline.

**Fig. 4:**
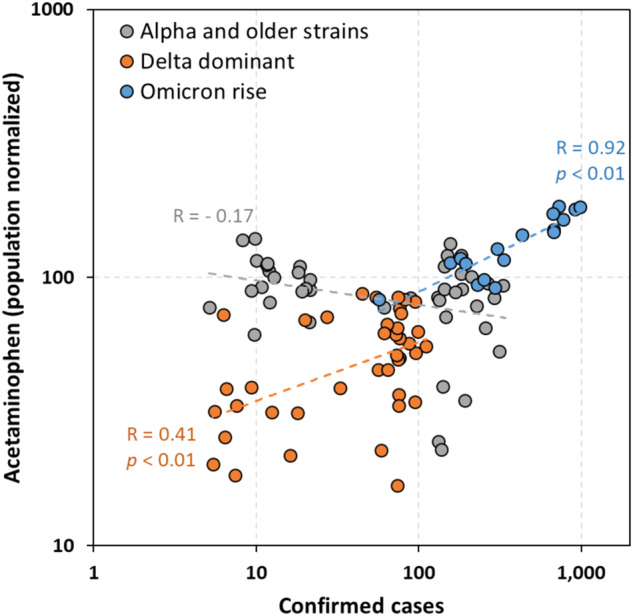
Correlations between the confirmed cases and acetaminophen. Three different stages of prevailing viral variants are presented in different colors.

The temporal dynamics of other pharmaceuticals in wastewater over time appear in Fig. 3. Among the 12 consistently detectable compounds (caffeine was excluded because it was used for population normalization), most of them had distinct individual patterns over time. Of these compounds, acetaminophen showed a very similar trend (Fig. 3a) to both the viral loads and confirmed cases, especially during the period when the Omicron variant was dominant. The estimated lead time of acetaminophen over the confirmed cases was 0–2 days, which is shorter than the lead time of the SARS-CoV-2 genes in wastewater. The timing of COVID-19 virus shedding from humans is thought to occur soon after infection [49, 50]. As patients develop symptoms, preliminary treatment (e.g., OTC drugs) might be taken to ease the symptoms of illness and then followed by diagnosis (i.e., confirmed cases). This sequence agrees with our observation. However, our results contrast with those of a study conducted in Western New York [30], which reported the peak of acetaminophen spiking at ~2.5 weeks before the peak of the virus detected in wastewater. This discrepancy may be related to residents’ behavior regarding their usage of OTC drugs between the two studied areas and/or a different length of the monitoring period analyzed. Our finding of a short-to-non-existent lead time (0-to-2 days) of acetaminophen and viral loads and reported cases seemingly is more consistent with the fact that COVID symptoms proceed to viral infection and presumably viral shedding by only a day [50]. We further broke down our trend line into three periods dominated by different SARS-CoV-2 variants and analyzed their correlation with acetaminophen and found that acetaminophen shows significant positive correlations with the confirmed cases when the delta variant was dominant and after the emergence of the Omicron variant (Fig. 4), affirming the robustness of acetaminophen as a wastewater marker of COVID-19 cases across the pandemic. The correlation for the alpha variant is not statistically significant, although the calculated slope is −0.17 (Fig. 4). One explanation is the start of vaccination in early 2021 in the NYS, which was around the transition time from the alpha-epsilon to the delta variants. We speculated that people started to use OTC drugs to treat mild symptoms after vaccination.

### Forecasting model using viral gene and biomarker data

**Fig. 5:**
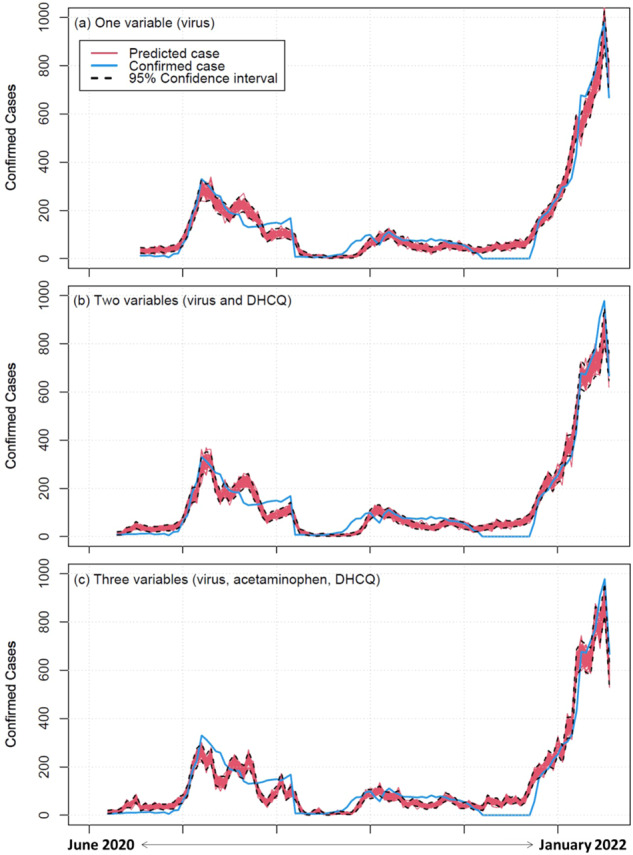
Confirmed case predictions using viral gene and biomarker data from June 2020 to January 2022. A different set of predictors is used in each panel: (**a**) virus, (**b**) virus and desethylhydroxychloroquine, and (**c**) virus, acetaminophen, and desethylhydroxychloroquine. The red lines and their respective black dashed lines in each panel represent the posterior predictions with a 95% confidence interval. The blue lines show the real confirmed cases.

Three models were built and fitted to data collected in Suffolk County, NY, USA from June 2020 to January 2022 (Fig. 5). Based on our data exploration results, three sets of predictors: viral gene copies, acetaminophen, and desethylhydroxychloroquine were used in the models (see the section of Model development in the SI for more details). In general, the convergence of the MCMC appeared healthy based on the diagnostics details in the SI, including potential scale reduction factor (PSRF), effective sample size (ESS), trace plots, and trace rank plots. The posterior distributions of parameters and their 95% intervals are shown in the SI, and the models’ posterior predictions are plotted against the data in Fig. 5a–c. The estimates from the models provide reasonable descriptions of the data, and allow us to reconstruct the temporal trends of the confirmed cases throughout the entire ~20 months of observation. When the estimation involved acetaminophen and desethylhydroxychloroquine (Fig. 5b, c), the predictions tracked the data better during the Omicron surge, presumably due to a very strong correlation between the confirmed cases and acetaminophen specifically at that stage (Fig. 4). Note, however, that these predicted time trends should not be perfectly aligned with collected data of confirmed cases to avoid overfitting and to have out-sample accuracy.

**Fig. 6:**
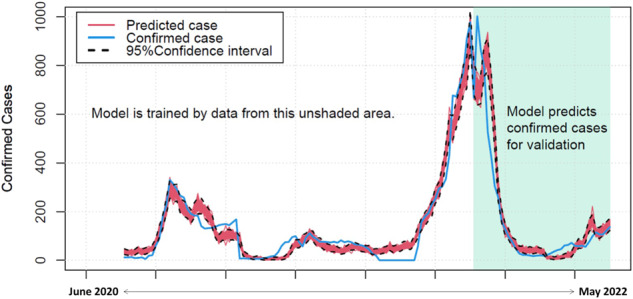
Model validation using ongoing viral data collected after January 2022 (shaded area). The model was developed and trained with the data collected from June 2020 to January 2022 (unshaded area).

We used an additional dataset of viral concentrations to validate our model. The data were collected from January 2022 through May 2022, consisting of 39 measurements. However, because analysis of COVID-treatment and OTC drugs were not available from this period, we were only able to validate our first model with the prediction solely by viral RNA concentrations. We used viral concentrations from this new dataset as inputs to predict confirmed cases in the same period (Fig. 6). It should be noted that this dataset was not used for model development. The shaded area in Fig. 6 indicates that the model captured the confirmed case trend quite well, with the exception of the peak in cases. The difference between the predicted and reported cases around the peak might be due to measurement uncertainties or other human factors. Nonetheless, considering the model can be continuously calibrated by new datasets and its purpose for out-sample prediction, it should have utility for future use.

### Study limitations

One major limitation for applying WBE during an emerging pandemic could be the immediate availability of analytical standards for treatment drugs and references for viral lineages, which is critical for rapid method development with accuracy and precision. For example, the isotopically labeled remdesivir was not commercially available during the time the analysis was performed in this study.

SARS-CoV-2 is also known to be associated with solid particulate and debris in wastewater, and monitoring viral signals on wastewater solids (e.g., sludge) has also been utilized as an indicator of COVID-19 outbreak in communities [51, 52]. In this study, our viral extraction method was developed and optimized for filtered wastewater. This may slightly underestimate the overall viral concentration in raw wastewater influent but would not alter the trends observed over time. Moreover, to detect particulate-associated viruses, sedimented sludge is reported to be a better option than suspended particles in the influent [51]. Collecting samples from aqueous and/or particulate phases for WBE applications depends on the nature and behaviors of analytes of interest in wastewater, and the easiness and representativeness (24-h composite sampling) of sampling approaches should also be considered.

The purpose of modeling and prediction is to use available data to infer the unknown. Out-of-sample possibilities are therefore considered in modeling to reduce the chances of overfitting. That said, there might be some limitations to the model. In a Bayesian context, these limitations can arise from mistaken assumptions about the underlying infection process, such as the distributions of parameters and the selection of priors. Measurement errors of substances within and across research sites, as well as human factors like state- or borough-wise policies, could undermine the accuracy of predictions. Fortunately, thanks to the flexibility of Bayesian models, new datasets from different areas and times can be utilized to calibrate the model.

## Conclusions

Monitoring of viral RNA, COVID-treatment drugs, and other pharmaceuticals in wastewater samples over a period of ~20 months in Suffolk County, NY, revealed that viral gene copies, across different variant prevailing periods, reflected the time series of COVID-19 confirmed cases in the sewer catchment area with a calculated lead time of 3–4 days. Antiviral drugs and their metabolites were detected with varying frequencies in wastewater samples. The rationale for monitoring COVID-19 treatment drugs in wastewater was to understand treatment of patients in the community. However, the stability of these drugs was low in wastewater and, hence, suggested that these drugs were not ideal biomarkers. However, acetaminophen (OTC) and desethylhydroxychloroquine were significant correlated with the viral concentrations in wastewater and acetaminophen was also correlated with the prevalence of COVID-19 in the community. Acetaminophen exhibited a short-to-non-existent lead time (0-to-2 days) ahead of the virus and reported cases, which agreed with the symptom progression of COVID-19. Acetaminophen is abundant in wastewater and can be analyzed with minimum sample preparation compared to viral RNA analysis. Since acetaminophen and other similar OTC drugs are not specific to COVID treatment, their variations in wastewater may inform important changes in population health within the sewershed. We suspect other viral outbreaks with similar symptoms may also be revealed by monitoring these OTC drugs in wastewater. Using the viral RNA and pharmaceuticals data, we developed Bayesian models to predict the confirmed cases (infected individuals) within the catchment area. The models were capable of reproducing the temporal trend of the confirmed cases from June 2020 to January 2022 and accurately predicting COVID-19 cases in the community using viral loads in wastewater from January to May 2022.

### Supplementary information

Supplementary Figures and Tables

Supplementary Information

## Data Availability

The data generated and analyzed in this study are available upon reasonable request.
