# Supplementary material for: Monitoring of over-the-counter (OTC) and COVID-19 treatment drugs complement wastewater surveillance of SARS-CoV-2
Source: J Expo Sci Environ Epidemiol. 2023 Dec 5;34(3):448–56. doi: 10.1038/s41370-023-00613-2 (PMC11222153; doi:10.1038/s41370-023-00613-2)
Supplement: Supplementary file 2 — Supplementary Information [file 41370_2023_613_MOESM2_ESM.docx]

**Supplementary Information**

**Model development**

**a. Data Exploration**

From correlation plots together with our prior knowledge, our variables of interest are shown below,

1. Confirmed cases (*C_t_*)

2. Virus (*V_t_*)

3. Acetaminophen (*A_t_*)

4. Desethylhydroxychloroquine (*DH_t_*)

**b. Cross Correlations**

We also look at cross-correlations between the confirmed cases and (1) virus, (2) acetaminophen, and (3) desethylhydroxychloroquine (see figures below). That is, if there is any lead time between the peaks of any of the three variables and the confirmed cases. From those results specifically, desethylhydroxychloroquine seems to be a good indicator for early warning of cases outbreak.


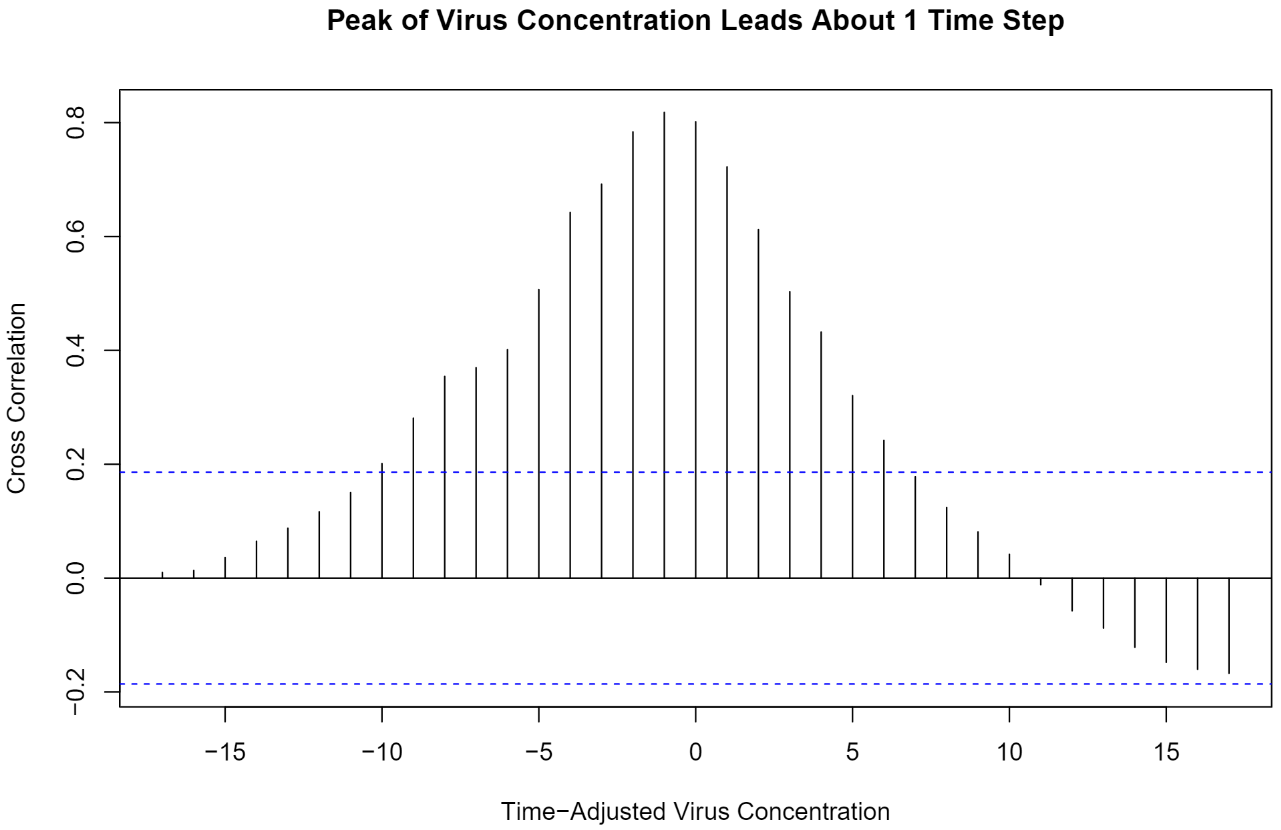

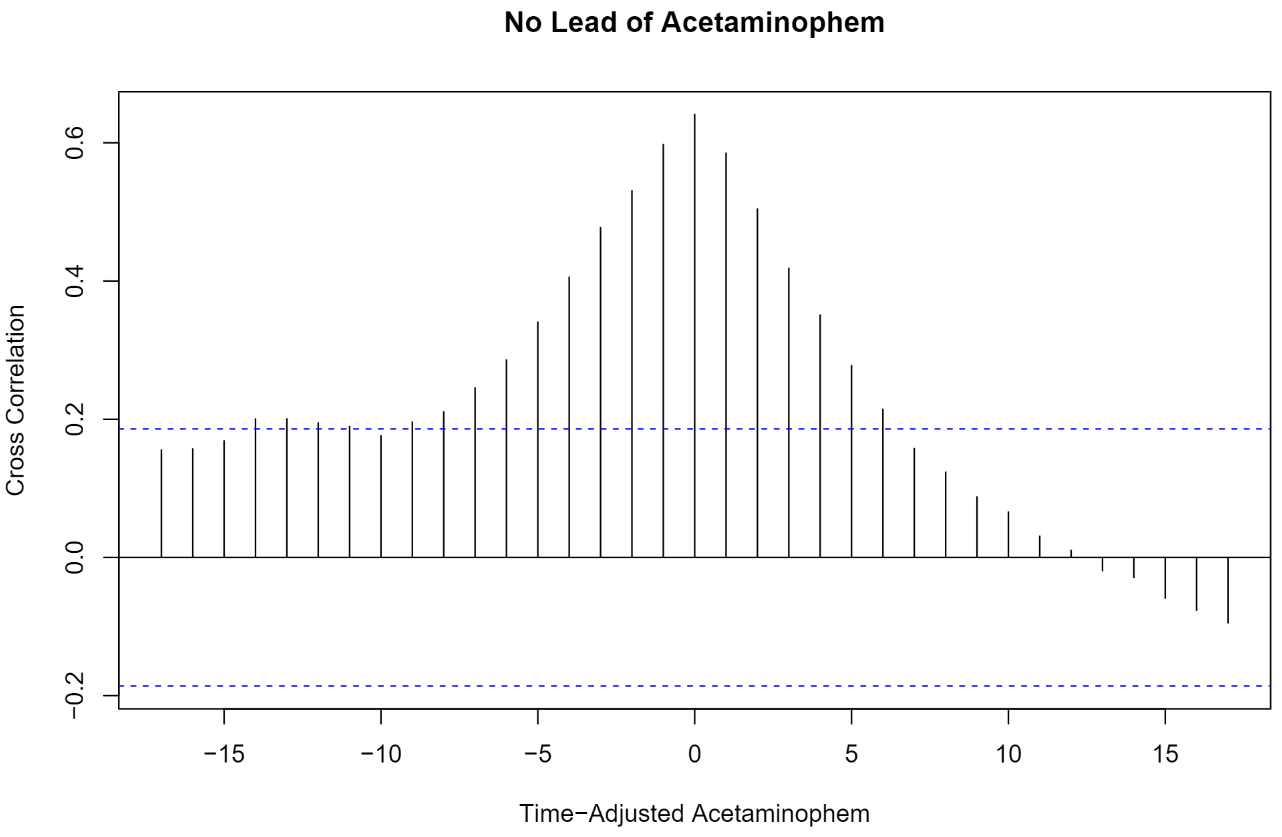

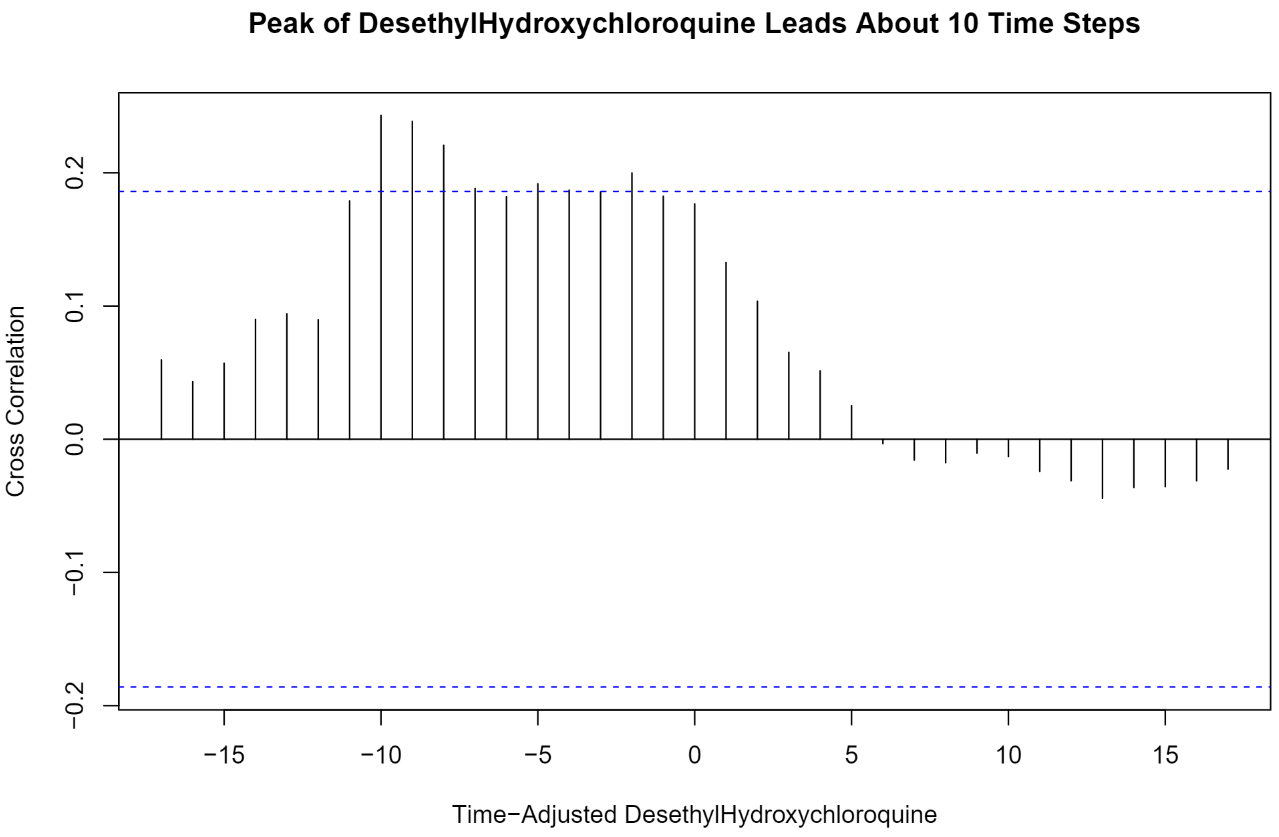


**c. Temporal Trends**

For time series plots of these variables, virus, acetaminophen, and desethylhydroxychloroquine show a similar trend to our outcome variable, confirmed case, and are chosen as predictors in our models

**d. Models**

Three specific sets of models are considered:
1. M-110s: models with one substance and ten lags (m = 1, n = 10).

2. M-205s: models with two substances and five lags (m = 2, n = 5).

3. M-303s: models with three substances and three lags (m = 3, n = 3).

For each set of models, we test possible combinations of variables according to Data Exploration and Cross Correlation. We retain the model with the best predictive performance based on the Watanabe–Akaike Information Criterion (WAIC).

*M-110s:*M-110s regress the current confirmed cases on ten lags of a single predictor variable (1 x 10). Candidate substances considered for this model: (1) virus, (2) acetaminophen, and (3) desethylhydroxychloroquine. The virus model has the lowest WAIC score and has the following form:


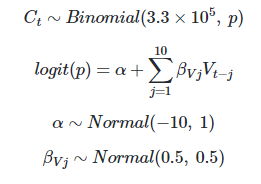


*M-205s:*
M-205s regress the current confirmed cases on five lags of two predictor substances (2 x 5). The model with the virus concentration and desethylhydroxychloroquine has the best performance and has the following form:


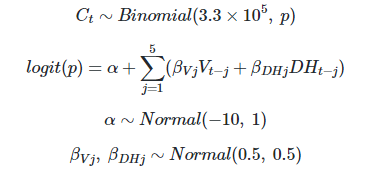


*M-303s:*
M-303s regress the current confirmed cases on three substances, each with three lags (3 x 3). The best model uses (1) virus, (2) desethylhydroxychloroquine, and (3) acetaminophen as predictors and has the following form:


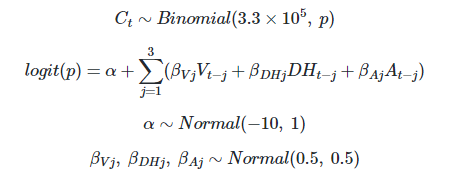


**e. Prior Predictive Simulations**

Common priors are applied to all parameters to regularize our estimate. The choice of priors is based on their resulted confirmed case distributions. For a specific set of priors, we calculate its infection probabilities (by taking the inverse logit of the linear combination of parameters) and the simulated confirmed cases (via a binomial process) under different distributions of virus concentration. As a result, the following set of priors is chosen:


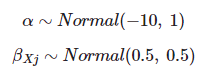


Here, we show how infection probability and confirmed cases are distributed under three cases of virus distributions.

*Case 1: virus concentration distributed uniformly*


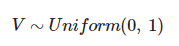


Distribution of Infection Probability

| **Min.** | **1st. Qu.** | **Median** | **Mean** | **3rd Qu.** | **Max.** |
| --- | --- | --- | --- | --- | --- |
| 0.0000016 | 0.0001322 | 0.0004810 | 0.0048370 | 0.0021727 | 0.4947711 |


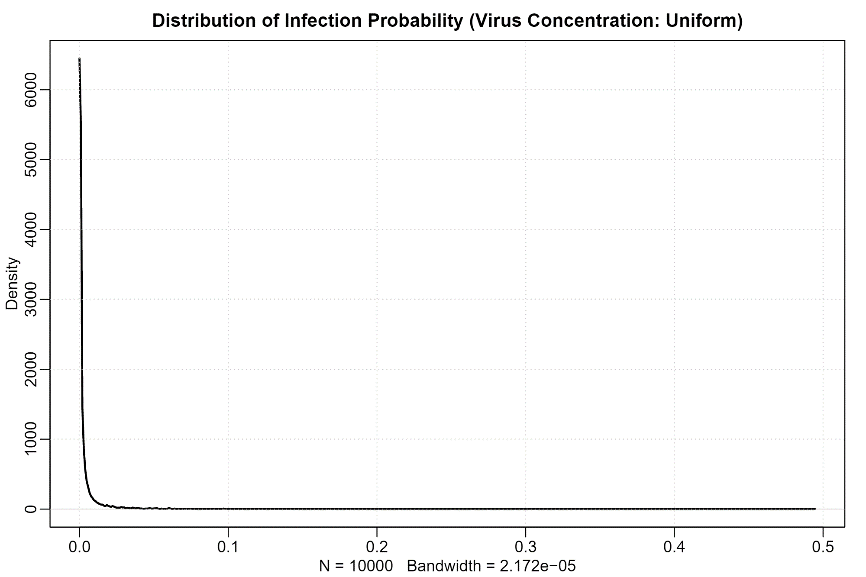


Distribution of Confirmed Case

| **Min.** | **1st. Qu.** | **Median** | **Mean** | **3rd Qu.** | **Max.** |
| --- | --- | --- | --- | --- | --- |
| 0 | 44 | 158 | 1596 | 715 | 163123 |


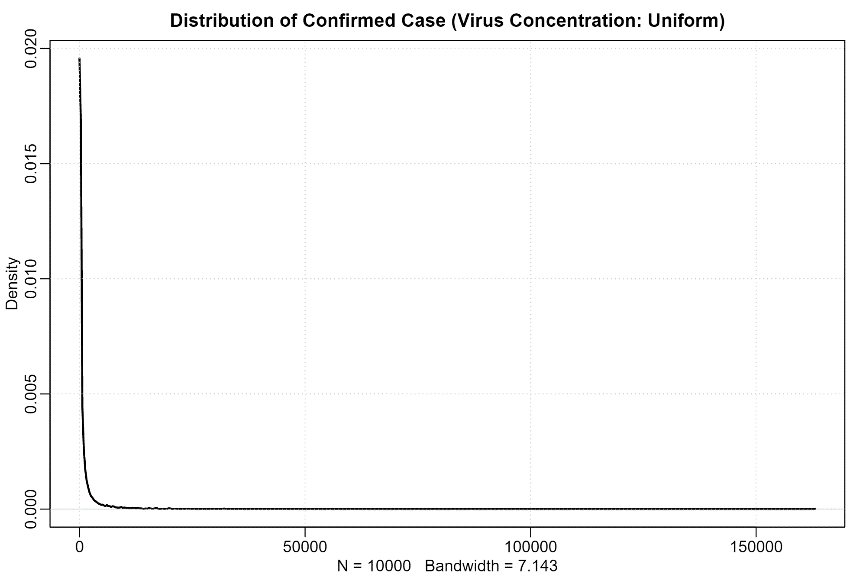


*Case 2: virus concentration at the maximum*


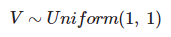


Distribution of Infection Probability

| **Min.** | **1st. Qu.** | **Median** | **Mean** | **3rd Qu.** | **Max.** |
| --- | --- | --- | --- | --- | --- |
| 0.0000094 | 0.0019102 | 0.0067741 | 0.0264734 | 0.0227059 | 0.8991982 |


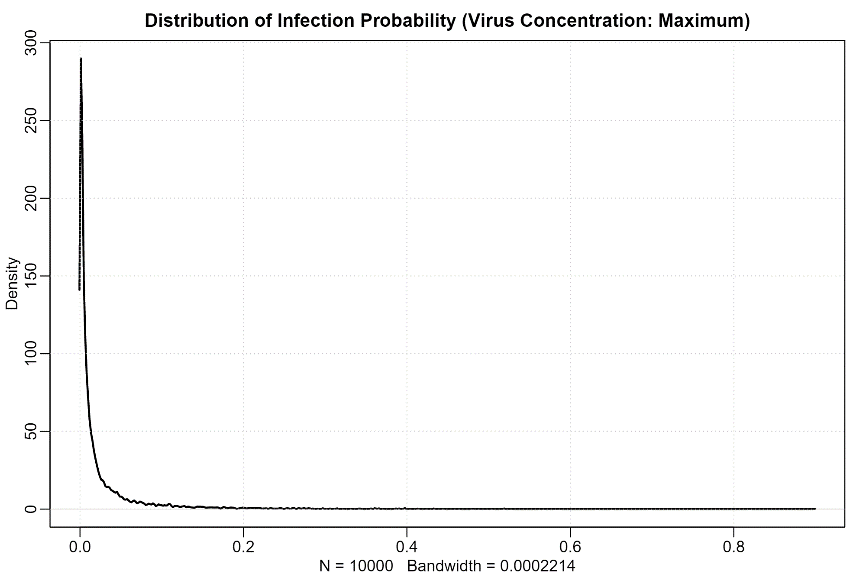


Distribution of Confirmed Case

| **Min.** | **1st. Qu.** | **Median** | **Mean** | **3rd Qu.** | **Max.** |
| --- | --- | --- | --- | --- | --- |
| 2 | 627 | 2240 | 8736 | 7501 | 296497 |


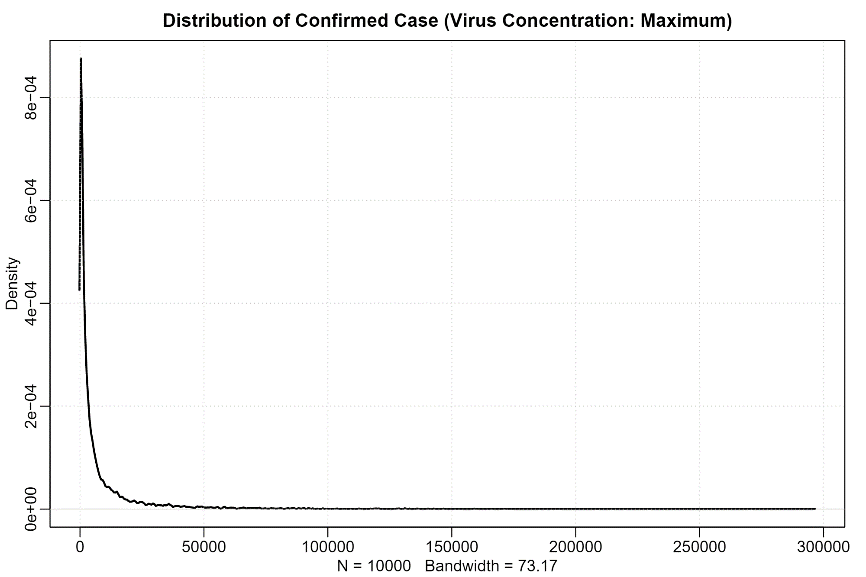


*Case 3: virus concentration at the minimum*


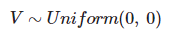


Distribution of Infection Probability

| **Min.** | **1st. Qu.** | **Median** | **Mean** | **3rd Qu.** | **Max.** |
| --- | --- | --- | --- | --- | --- |
| 9.011e-07 | 2.306e-05 | 4.538e-05 | 7.407e-05 | 8.776e-05 | 1.576e-03 |


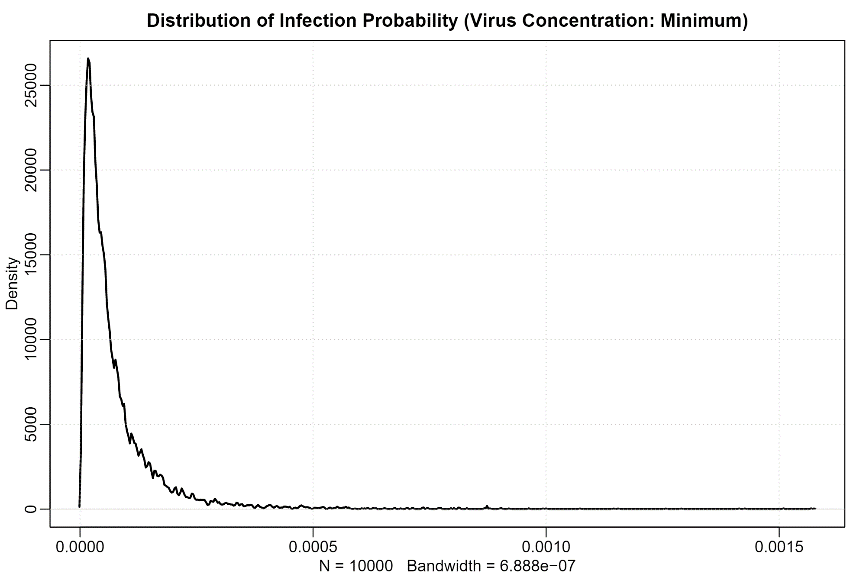


Distribution of Confirmed Case

| **Min.** | **1st. Qu.** | **Median** | **Mean** | **3rd Qu.** | **Max.** |
| --- | --- | --- | --- | --- | --- |
| 0 | 7 | 15 | 25 | 30 | 542 |


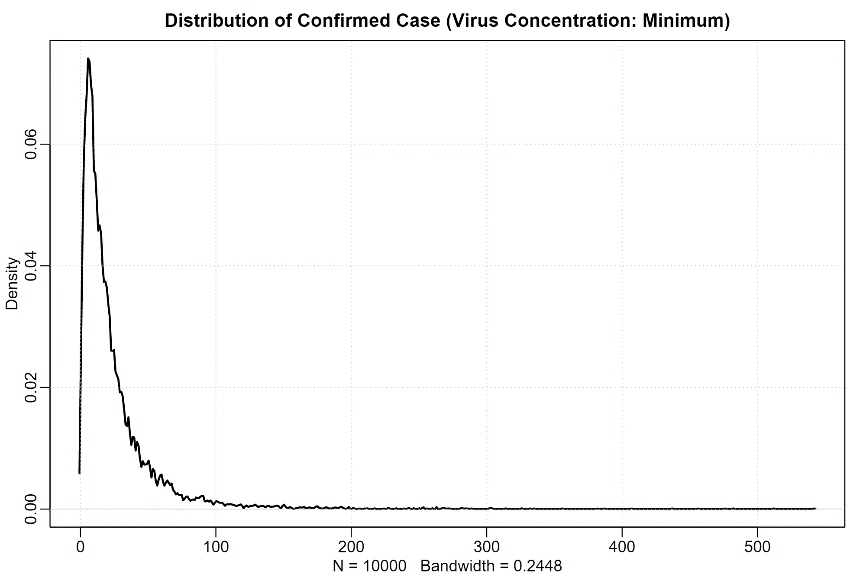


**f. MCMC Diagnostics**

Several criteria are used to gauge the sampling quality of the MCMC algorithm. Generally, for accurate inference, we look for three qualities of our Markov chain: (1) convergence, (2) stationary, and (3) good mixing. The trace and rank plots (Vehtari et al 2019) below show these features. The R-hats are close to one, and hence confirm the convergence (Brook and Gelman 2012). The ESSs (the effective MCMC length without autocorrelation) seem reasonable given the non-skewed posterior distributions shown in the Posterior Distributions and Coefficient Intervals section below. The means, standard deviations, and 95% percentile intervals of the posterior distributions for parameters are also provided.

*M-110s: Virus*R-hat and ESS table:

|  | **mean** | **sd** | **2.5%** | **97.5%** | **n_eff** | **Rhat4** |
| --- | --- | --- | --- | --- | --- | --- |
| a | -20.2427857 | 0.11476878989114 | -20.4620425 | -20.013475 | 2214.4863524851 | 1.00116317885218 |
| bV1 | 2.976528885 | 0.171112402199256 | 2.643832 | 3.31255975 | 1643.73214728439 | 1.00042066324287 |
| bV2 | 2.479392245 | 0.196401317925699 | 2.1000095 | 2.865888 | 1370.17549874953 | 1.0029853747985 |
| bV3 | 3.209276455 | 0.210821438767752 | 2.78020025 | 3.6148165 | 1490.86948641381 | 0.999423506131243 |
| bV4 | 2.75133813 | 0.199930503449593 | 2.362772 | 3.1503055 | 1654.00425493691 | 1.00076036118224 |
| bV5 | 1.1288664655 | 0.203925305643139 | 0.73637725 | 1.50726375 | 1429.28423218 | 1.00061958324148 |
| bV6 | 0.50851240765 | 0.191145413027201 | 0.15102115 | 0.888727625 | 1329.22964981724 | 0.999989500610677 |
| bV7 | 0.7954933373 | 0.203546563920791 | 0.379082925 | 1.195953 | 1504.67971532945 | 1.00041903592141 |
| bV8 | 1.168763591 | 0.209639824620233 | 0.752779125 | 1.59919675 | 1542.02061138882 | 1.00078714463858 |
| bV9 | 0.1405384286705 | 0.185773004216639 | -0.229559775 | 0.4951723 | 1288.99129650425 | 1.00108498177418 |
| bV10 | -0.473194798 | 0.175532874113711 | -0.79649015 | -0.11700585 | 1574.32602177099 | 0.999769590091101 |

Trace plot:


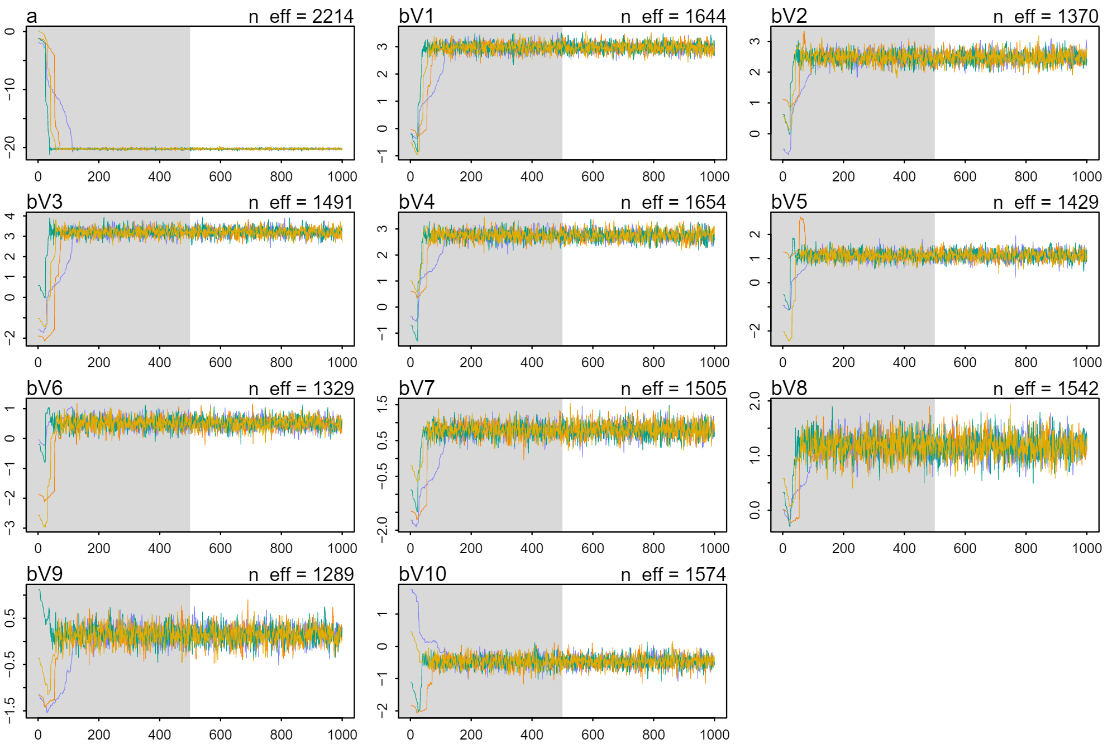


Rank plot:


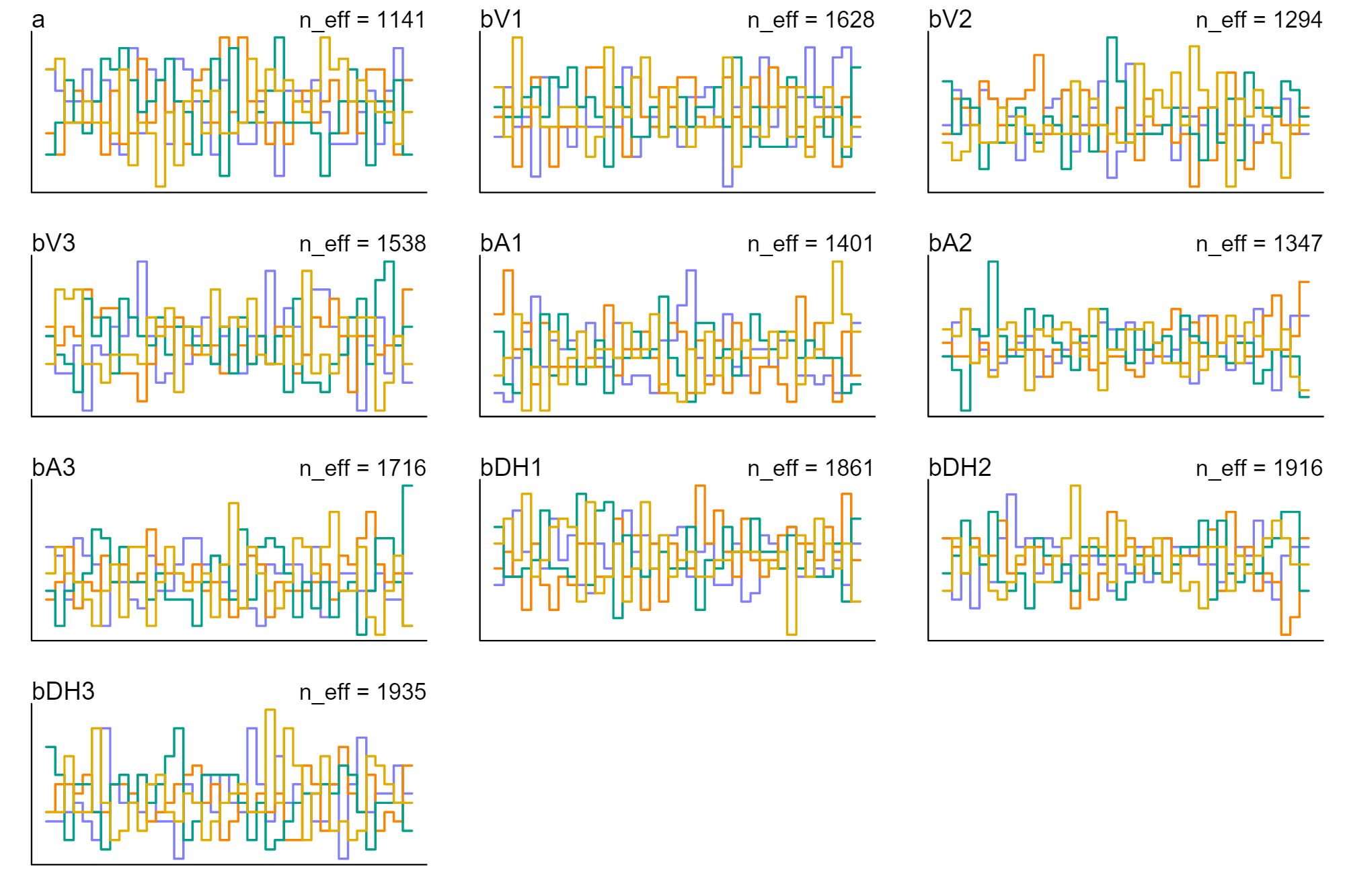


*M-205s: Virus, DHCQ*R-hat and ESS table:

|  | **mean** | **sd** | **2.5%** | **97.5%** | **n_eff** | **Rhat4** |
| --- | --- | --- | --- | --- | --- | --- |
| a | -20.45360485 | 0.113687392674404 | -20.6705225 | -20.23459 | 2311.38544786293 | 0.998826853006411 |
| bV1 | 3.05481166 | 0.184079955961636 | 2.68427175 | 3.41571275 | 1868.81050851382 | 0.999051632575679 |
| bV2 | 2.94109687 | 0.190333897299808 | 2.56817725 | 3.31896925 | 1800.32641132757 | 1.00098269638083 |
| bV3 | 3.606532705 | 0.197466960487001 | 3.23139575 | 3.98984925 | 1953.69907238804 | 0.999849399505337 |
| bV4 | 3.452917565 | 0.19594375253475 | 3.06231875 | 3.838581 | 1708.78515431058 | 1.00092232182518 |
| bV5 | 1.91873244 | 0.183183758141845 | 1.56771475 | 2.26638775 | 1484.89782391102 | 1.00061573922292 |
| bDH1 | -0.076415091929 | 0.0614733536293056 | -0.200453525 | 0.0433177524999999 | 2746.80363408721 | 0.99885262344014 |
| bDH2 | 0.1092584038235 | 0.054669528693183 | 0.00272403975000003 | 0.213536225 | 2014.83751424707 | 0.999782270906632 |
| bDH3 | -0.3860911695 | 0.0547408185972599 | -0.490672975 | -0.28253515 | 1993.42221739312 | 1.000146310914 |
| bDH4 | -0.30162288 | 0.0492771349498328 | -0.39849395 | -0.20997505 | 2272.54692022841 | 0.998717756238328 |
| bDH5 | 0.25627089765 | 0.0501226627452254 | 0.157081075 | 0.355773675 | 2801.84521842147 | 0.999839724174783 |

Trace plot:


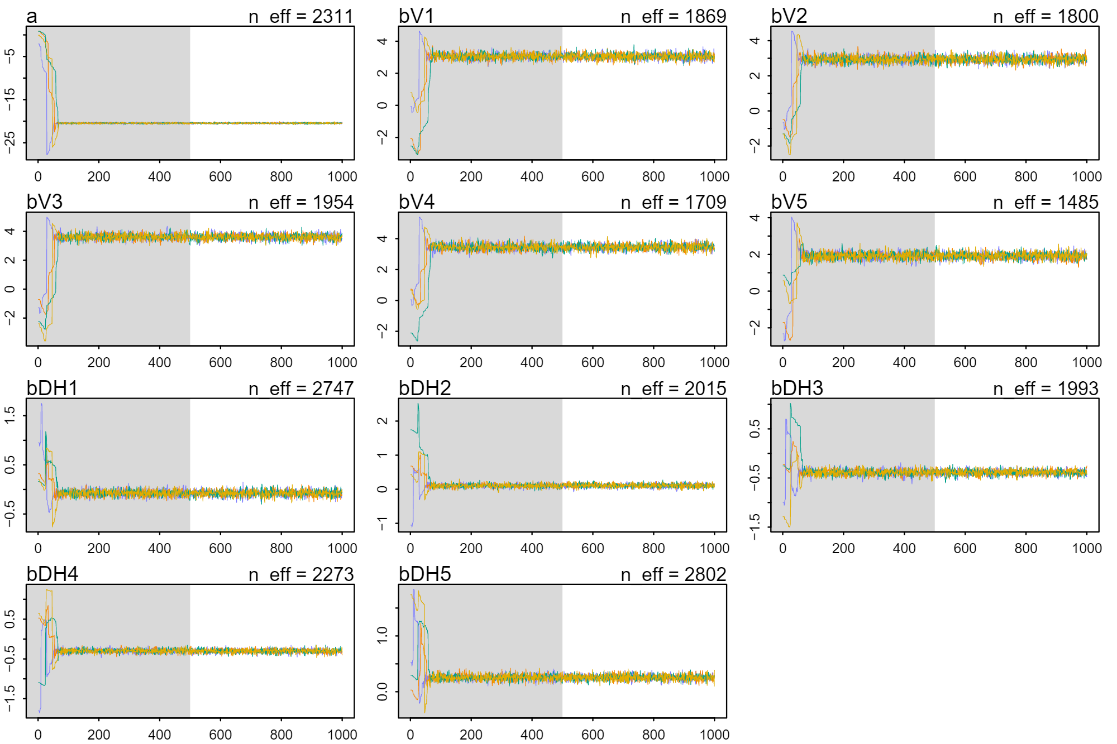


Rank plot:


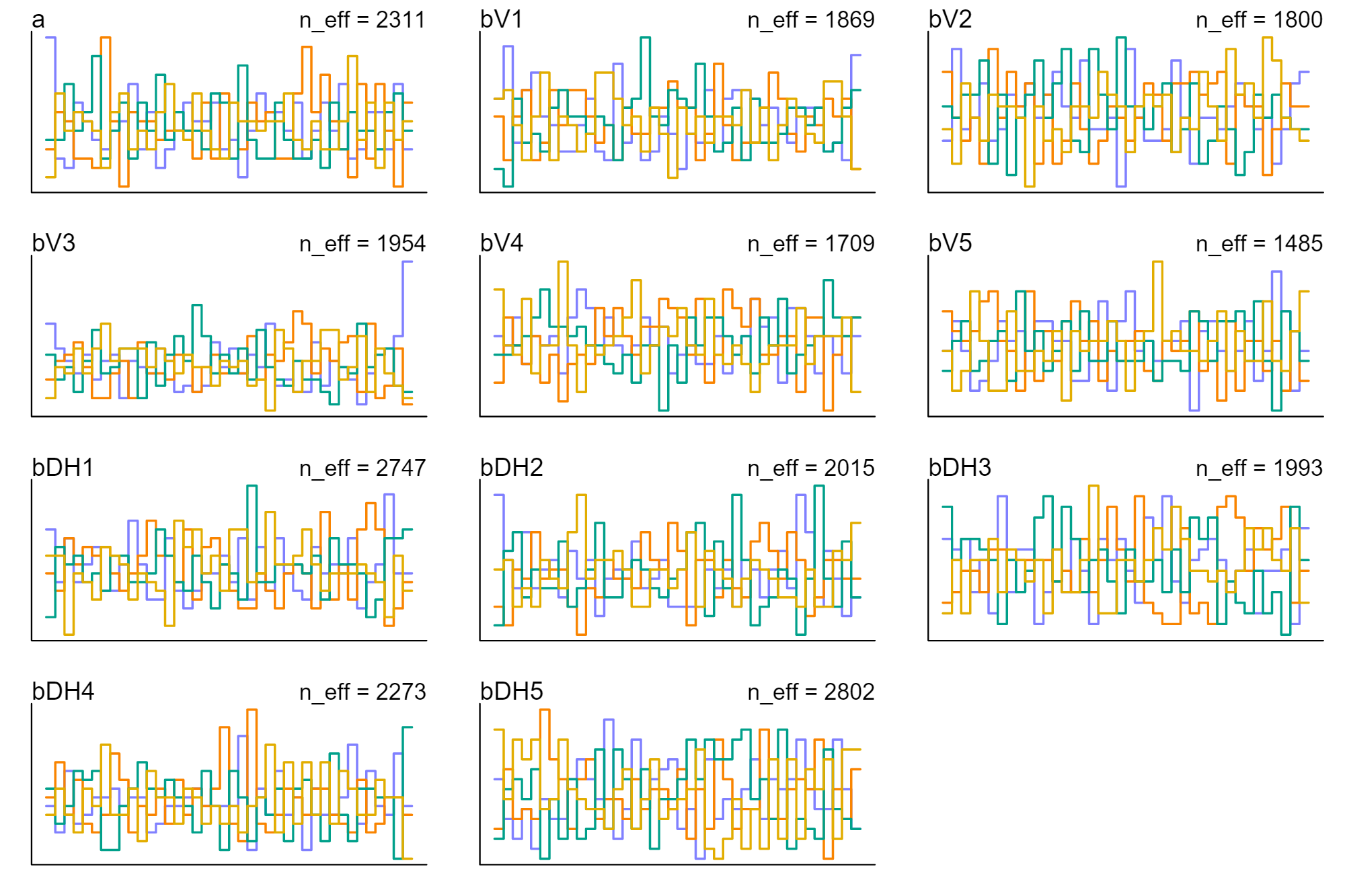


*M-303s: Virus, Acetaminophen, DHCQ*R-hat and ESS table:

|  | **mean** | **sd** | **2.5%** | **97.5%** | **n_eff** | **Rhat4** |
| --- | --- | --- | --- | --- | --- | --- |
| a | -18.91869605 | 0.173812719471627 | -19.25225 | -18.580265 | 1140.60054647012 | 0.999667279050107 |
| bV1 | 3.555158465 | 0.174897765423601 | 3.2312505 | 3.901155 | 1628.39388147896 | 0.999430556485637 |
| bV2 | 4.18278649 | 0.190557405516496 | 3.807111 | 4.56528 | 1293.77095374366 | 1.00169140368818 |
| bV3 | 5.091493575 | 0.186956001008328 | 4.7366525 | 5.45865125 | 1538.02841990903 | 1.00117099851413 |
| bA1 | -0.0454128706412 | 0.0692575847326355 | -0.1803886 | 0.0869741625 | 1401.33249140676 | 1.00183336390553 |
| bA2 | 0.17687891344 | 0.0715849341741194 | 0.0367496225 | 0.319598775 | 1346.94086469418 | 1.00263185466211 |
| bA3 | 0.671696725 | 0.0646138993655002 | 0.542563975 | 0.792891525 | 1715.88595300365 | 0.99974900042454 |
| bDH1 | -0.3279348945 | 0.0608648413996099 | -0.44648045 | -0.2092736 | 1861.25212269855 | 1.00282111811401 |
| bDH2 | -0.16147877917 | 0.0555688817119925 | -0.271931225 | -0.055911375 | 1916.20468056737 | 1.00025738625242 |
| bDH3 | -0.295262143 | 0.0550883493838168 | -0.4022526 | -0.18791735 | 1934.80854984805 | 0.999810933974121 |

Trace plot:


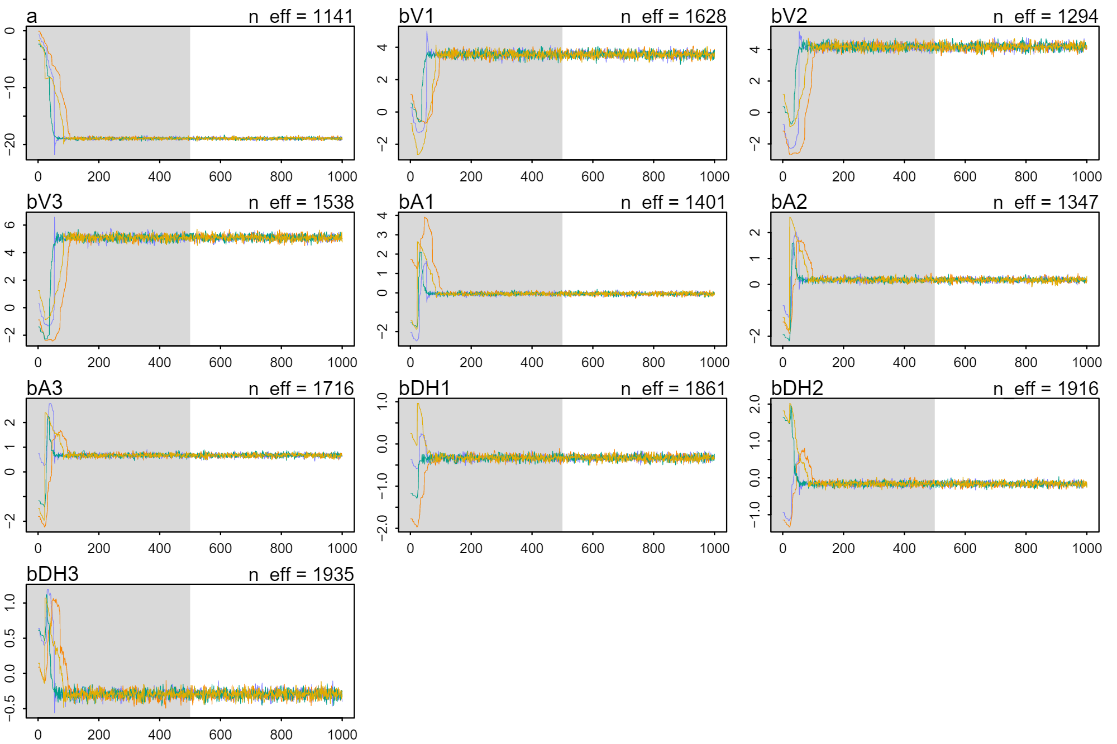


Rank plot:


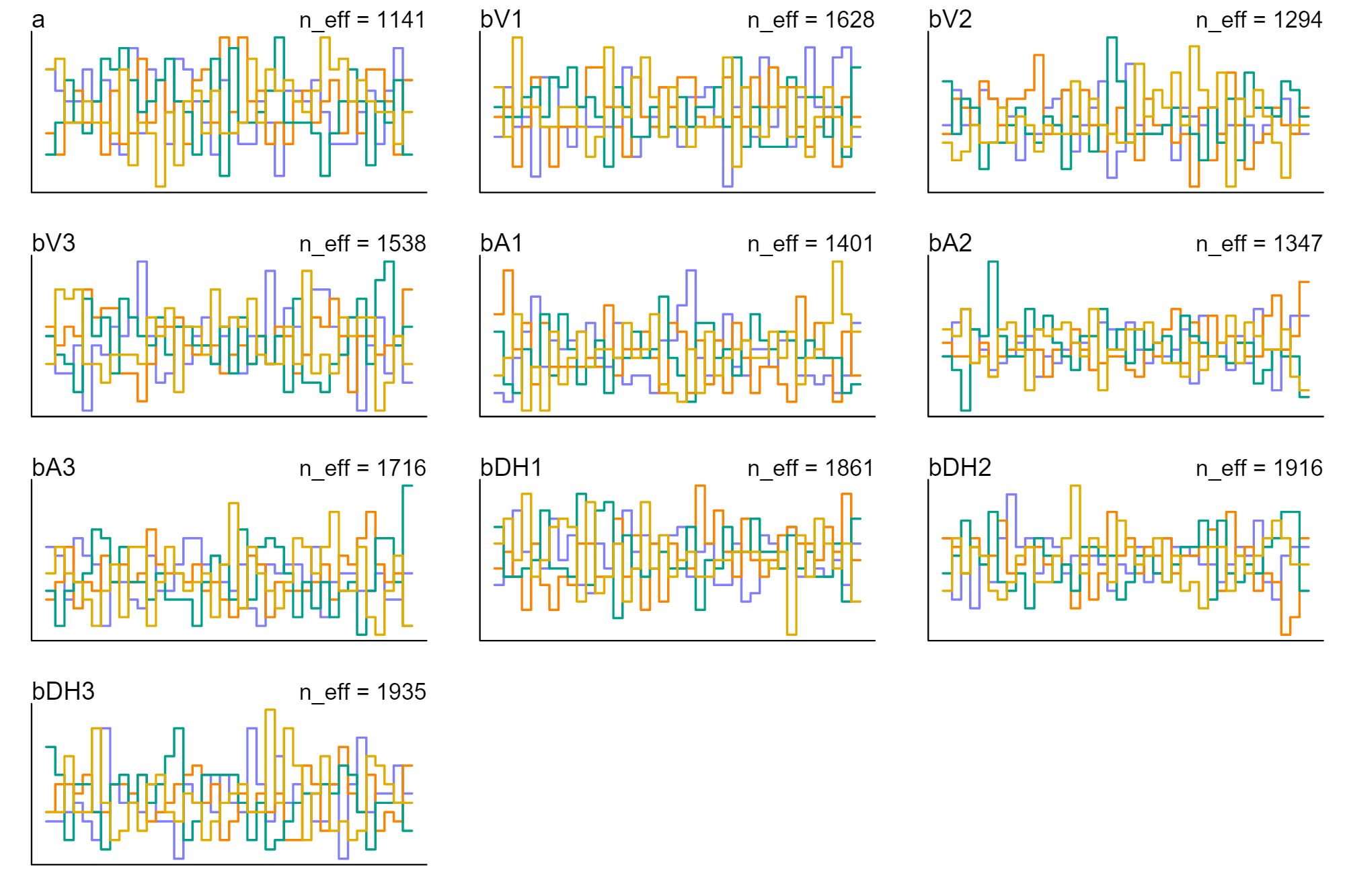


**g. Posterior Distributions and Coefficient Intervals**

The posterior distributions of parameters and their 95% intervals are shown below.

*M-110s: Virus*Distributions:

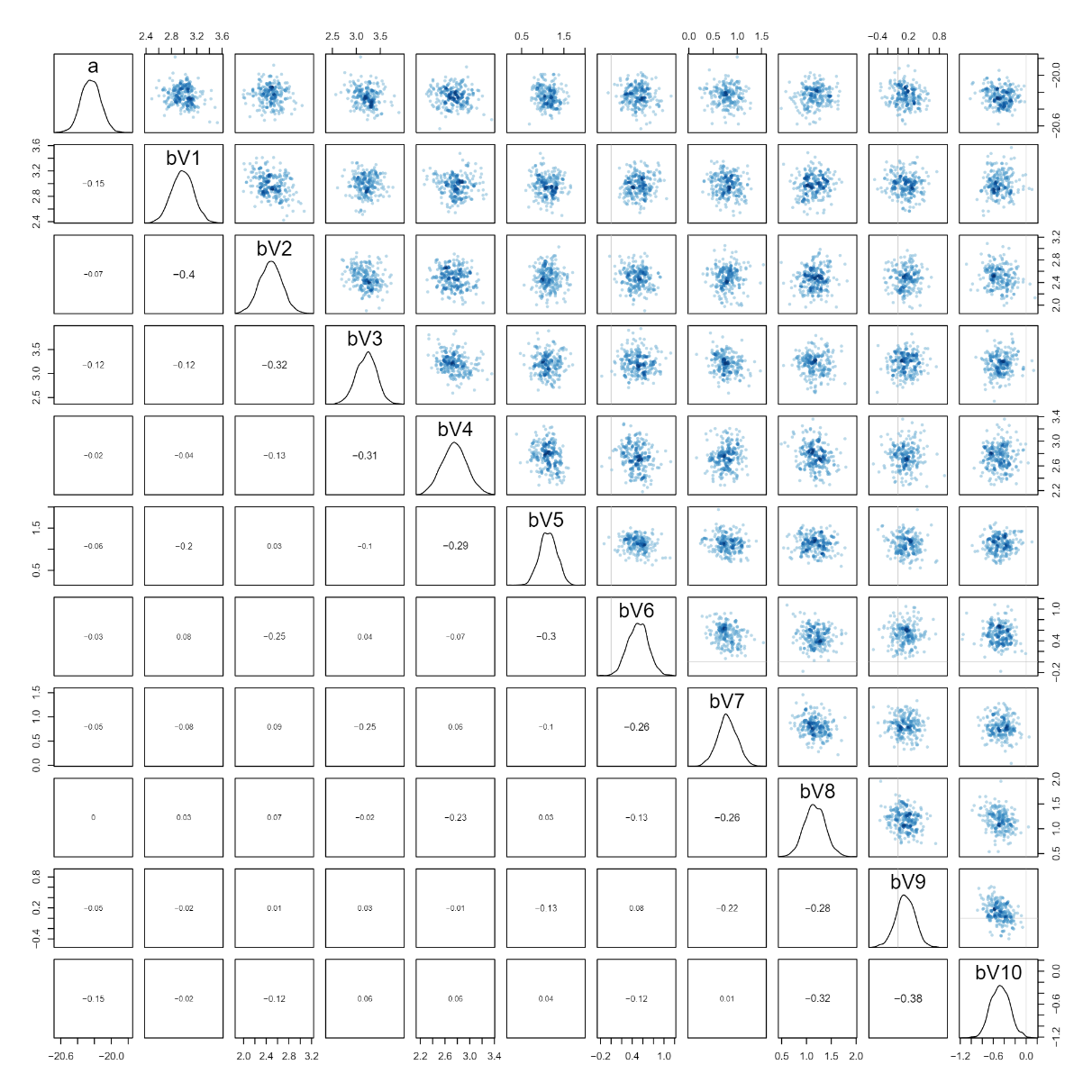


Coefficient Intervals:

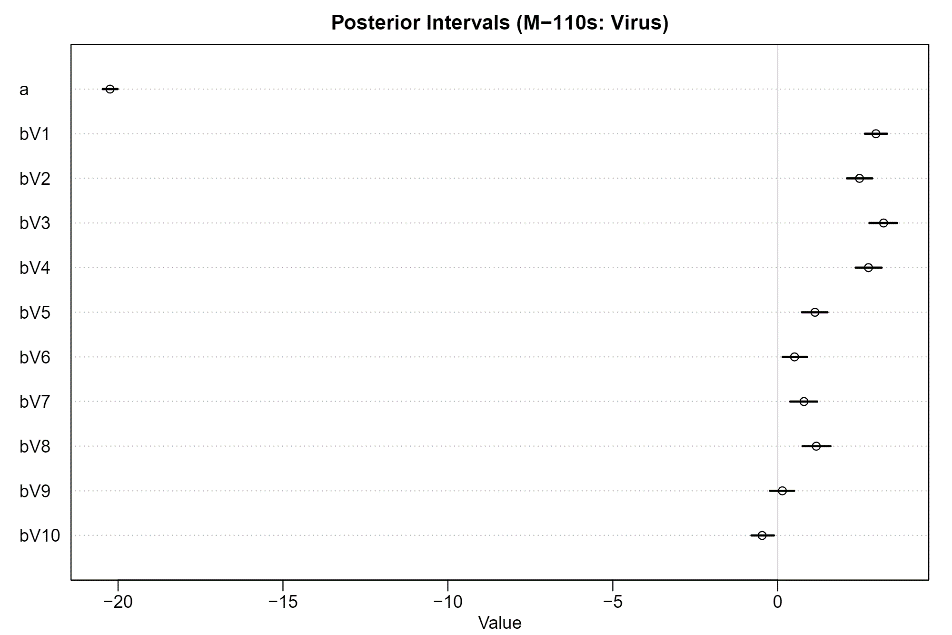


*M-205s: Virus, DHCQ*Distributions:

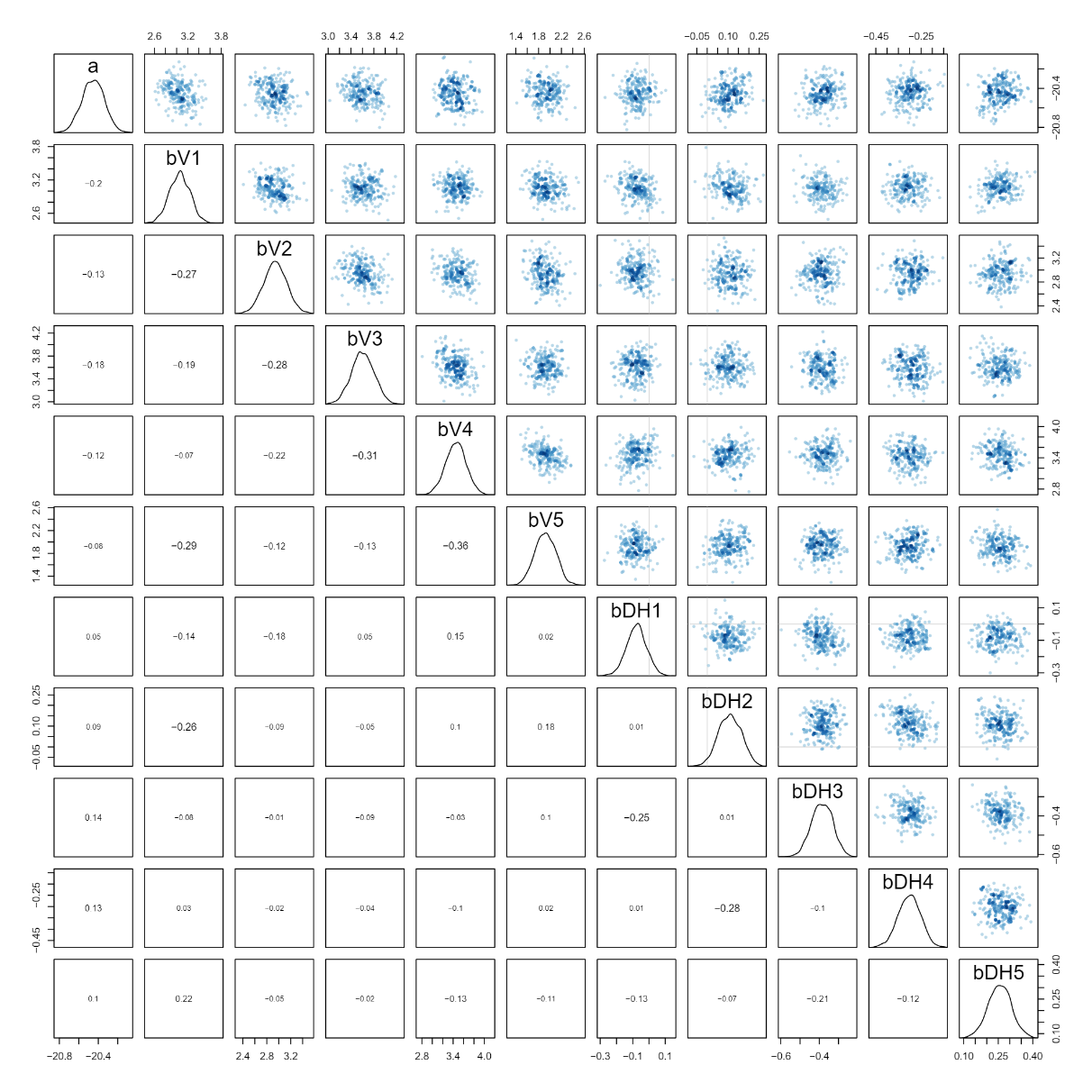

Coefficient Intervals:

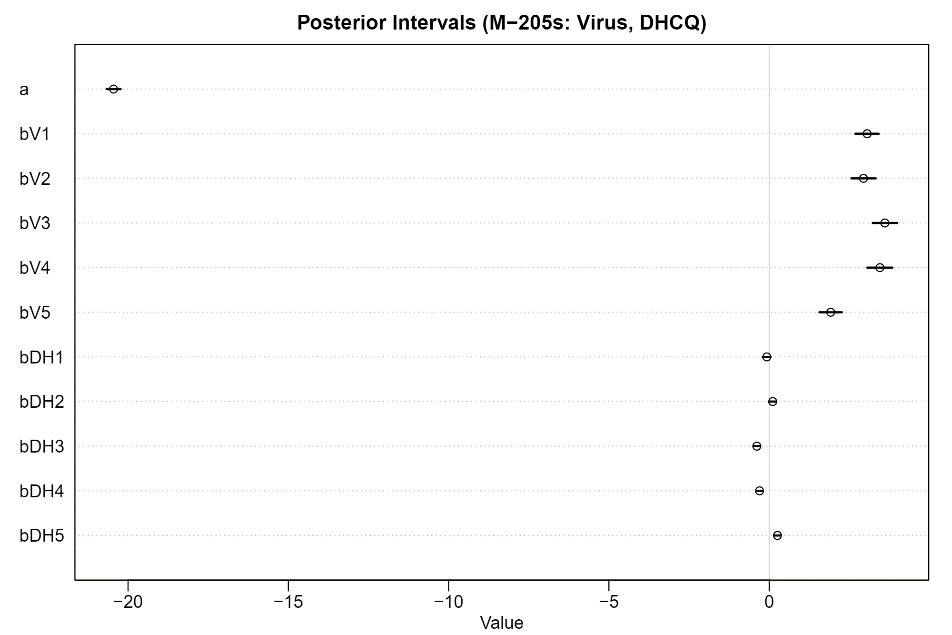


*M-303s: Virus, Acetaminophen, DHCQ*Distributions:

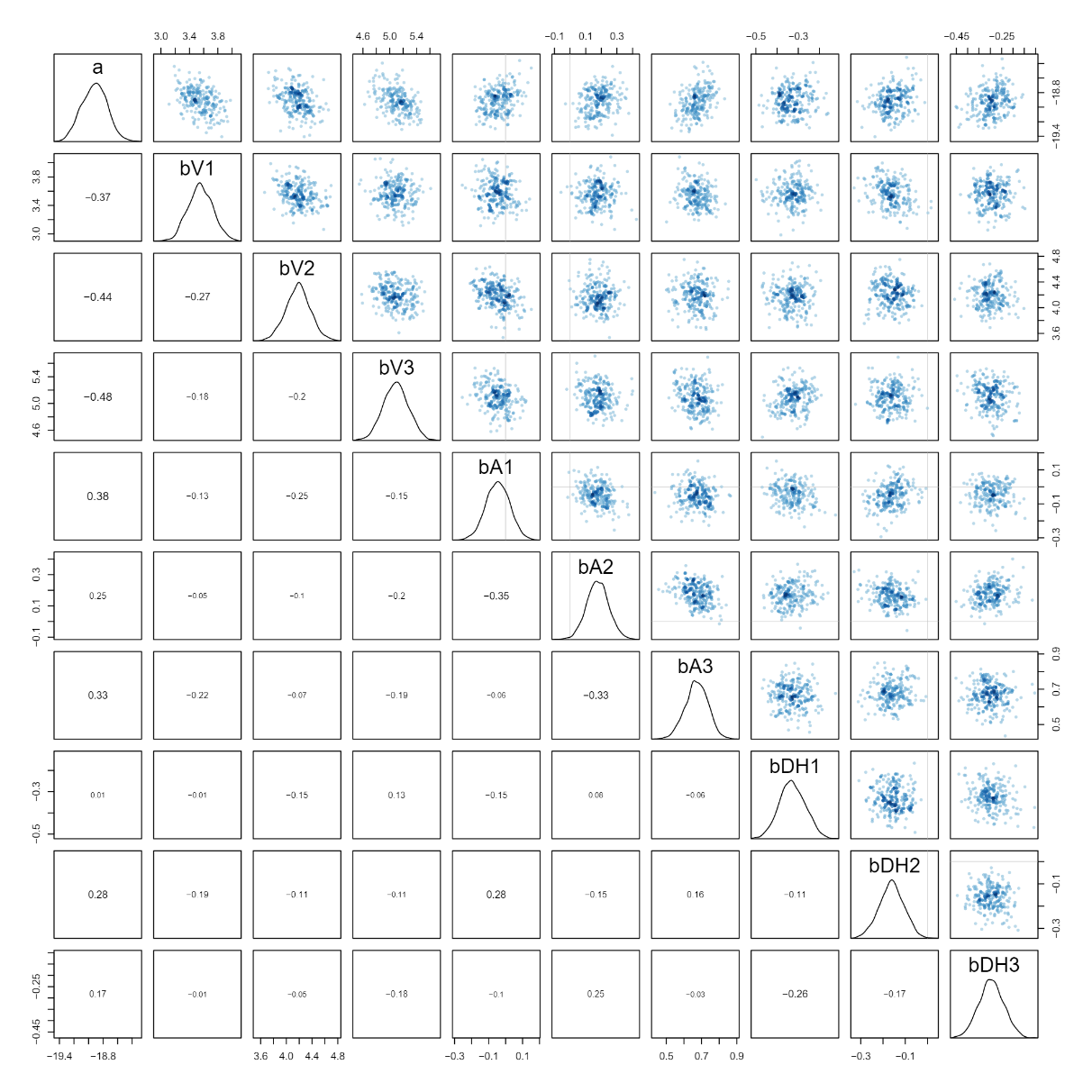

Coefficient Intervals:

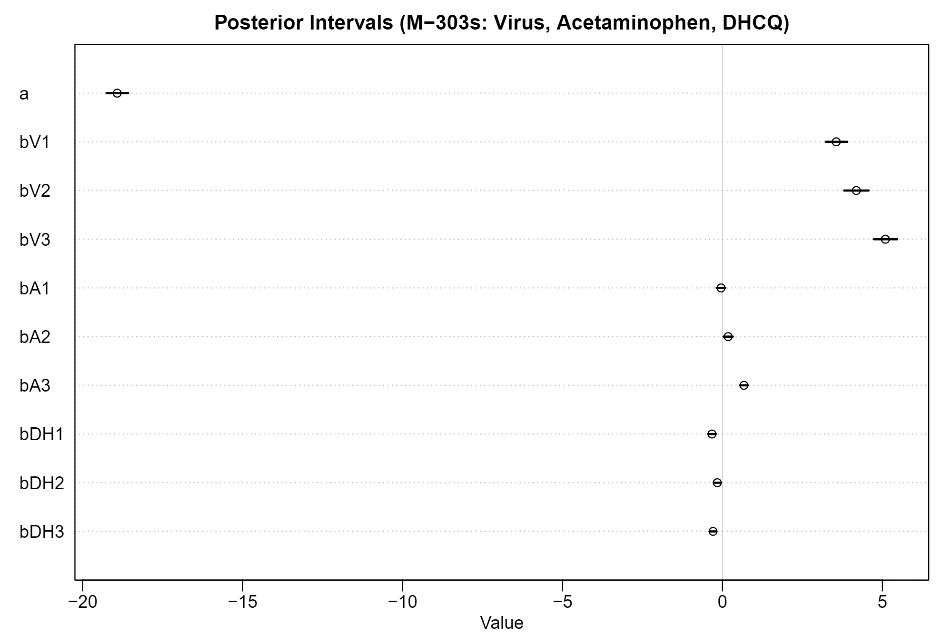


**Reference**

Vehtari, A., Gelman, A., Simpson, D., Carpenter, B., & Bürkner, P. C. (2021). Rank-normalization, folding, and localization: An improved R ̂ for assessing convergence of MCMC (with discussion). *Bayesian analysis*, *16*(2), 667-718.

Brooks, S. P., & Gelman, A. (1998). General methods for monitoring convergence of iterative simulations. *Journal of computational and graphical statistics*, *7*(4), 434-455.
